# Hem1 controls T cell activation, memory, and the regulated release of immunosuppressive and proinflammatory cytokines

**DOI:** 10.1172/jci.insight.174235

**Published:** 2025-07-08

**Authors:** Alexandra Christodoulou, Nutthakarn Suwankitwat, Jacob T. Tietsort, Ryan Culbert, Julia Y. Tsai, Fatima Tarbal, Chengsong Zhu, Brian M. Iritani

**Affiliations:** 1 Department of Comparative Medicine, University of Washington, Seattle, Washington, USA.; 2 Department of Immunology, Microarray and Immune Phenotyping Core Facility, University of Texas Southwestern Medical Center, Dallas, Texas, USA.

**Keywords:** Immunology, Inflammation, Adaptive immunity, Cytoskeleton, T cells

## Abstract

Hematopoietic protein-1 (Hem1) is a component of the WASp family verprolin-homologous protein (WAVE) actin regulatory complex, which is activated downstream of multiple immune receptors. Mutations in the *NCKAP1L* gene encoding HEM1 have recently been found to result in severe primary immunodeficiency disease (PID), characterized by recurrent respiratory infections, hyperinflammation, autoimmunity, and high mortality. However, how loss of Hem1 results in PID is unclear. To define the importance of Hem1 specifically in T cells, we generated constitutive and T cell–specific Hem1-null mice. Hem1-deficient T cells exhibited an increased shift from naive to memory T cells and increased ratio of immunosuppressive regulatory to effector T cells. Loss of Hem1 resulted in hallmarks of T cell exhaustion, including T cell lymphopenia, decreased activation and proliferation, increased expression of PD-1 and Tim3, and increased IL-10 production. In vitro TCR stimulation of CD4^+^ T cells resulted in increased production of Th1 (IFN-γ), Th2 (IL-5, IL-13), Th17 (IL-17, IL-22), and Treg (IL-10) cytokines. This correlated with reduced F-actin, increased expression of CD107a, and increased granzyme release indicative of increased granule membrane fusion and exocytosis. These results suggest that Hem1 is critical for maintaining T cell activation, homeostasis, and regulated cytokine production following antigen encounter.

## Abstract 2

T cell specific disruption of Hem1 results in impaired CD4 T cell activation, homeostasis, and the dysregulated release of Th1, Th2, Th17, and Treg cytokines

## Introduction

Inborn errors of immunity (IEIs) are typically caused by germline variants in single genes, which present clinically as increased susceptibility to infections, inflammatory diseases, allergy, autoimmunity, and in some cases malignancy (see ref. 1 for a review). In recent years there has been a steep increase in the number of causative genes identified in IEIs, driven by an increase in accessibility and affordability of next-generation sequencing technologies and improved ability to rapidly test the consequences of gene functions of specific gene variants using CRISPR and transgenic technologies. Among the novel IEIs recently described include 3 reports describing 9 individuals from 7 independent kindreds with severe primary immunodeficiency disease (PID) due to loss-of-function (LOF) mutations in the *NCKAP1L* gene encoding the actin regulatory protein hematopoietic protein-1 (Hem1) (2–4). Hem1-deficient children presented clinically within the first year of life with severe immunodeficiency characterized by recurrent bacterial and viral infections, otitis media, pneumonia, abscesses, cellulitis, septic arthritis, and gastroenteritis (see refs. 5, 6 for review). Autoimmunity and hyperinflammation were also commonly seen manifesting as immune complex glomerulonephritis, increased anti-nuclear antibodies and anti–double-stranded-DNA autoantibodies, and systemic lupus erythematosus–like disease. Hepatosplenomegaly and microcytic anemia with anisopoikilocytosis were also reported. Notably, siblings of patients from 3 of the 7 kindreds died prior to 3 years of age, emphasizing the severity of Hem1 deficiency and the importance of understanding the mechanisms underlying how loss of Hem1 predisposes children to infections and autoimmunity.

Hem1 is a hematopoietic cell–specific component of the WASp family verprolin-homologous protein (WAVE) actin regulatory complex (WRC), which acts downstream of multiple immune receptors to initiate actin nucleation in response to receptor stimulation (see refs. 6–8 for review). The WRC is a heteropentameric intracellular complex consisting of CYFIP1/2, WAVE1/2/3, HSPC300, ABI1/2/3, and Hem1/2 proteins that exists in an autoinhibitory state in resting cells. Following receptor stimulation, the small guanosine triphosphatases (GTPase) Rac1 and/or Arf1 are activated in the GTP-bound state, which then permits interactions with the inactive WRC, promoting association with the actin regulatory protein 2/3 (ARP2/3) nucleation complex. The ARP2/3 complex then drives nucleation of globular actin (G-actin) monomers into linear or branching F-actin bundles to generate filopodia and lamellipodia, which are important for many active processes in immune cells, including cell spreading, chemosensation, endo- and exocytosis, immune synapse formation, migration, and phagocytosis. Several IEIs associated with variants in genes involved with regulating the actin cytoskeleton have been described, further solidifying the importance of properly regulated actin polymerization in immune function (see ref. 8 for a review). Interestingly, the phenotypes of immune-related “actinopathies” are often unique due in part to the complexity of signaling pathways controlling actin remodeling, the numerous molecular players involved, the specific immune cell types impacted, and the intracellular locations affected by each mutation. Therefore, careful, detailed analyses of individual immune and nonimmune cell types using cellular assays and animal models are necessary to define the “whole body” consequences of gene variants in IEI.

We had previously utilized a “forward genetic, phenotype-driven” *N*-ethyl-*N*-nitrosourea chemical mutagenesis strategy in mice to identify novel genes involved in the development and functioning of the immune system. During the course of this program, we identified a pedigree with decreased B220^+^ cells and increased CD11c^+^ cells in peripheral blood (PB), which was mapped to a single noncoding point mutation in the *Nckap1l* gene encoding Hem1 (9). Similar to human patients with PID, mice with point mutations in Hem1 mice were severely immunodeficient, and were characterized by dwarfism, hepatomegaly, microcytic anemia, extramedullary hematopoiesis, reduced T and B cell development, altered T cell cytokine production, impaired phagocytosis, and impaired neutrophil migration (9–11). In addition, hyperinflammation and autoimmunity were noted, including glomerulonephrosis, amyloid deposition on liver margins, endocarditis, pancreatitis, colitis, typhilitis, and increased autoantibodies (3, 6, 9). However, because Hem1-point-mutated mice exhibited increased sensitivity to infections and failure to thrive, it was difficult to separate primary cell-autonomous effects from secondary effects following loss of Hem1. Thus, in this study, we generated T cell–specific Hem1-deficient mice using the Cre-LoxP system to define how loss of Hem1 impacts T cell development and function. We found that loss of Hem1 resulted in impaired T cell homeostasis, which manifested as reduced cell number, decreased proliferation and activation, and increased cytokine and granzyme release, which progressed to an exhausted-like memory phenotype. These results demonstrate how loss of Hem1, specifically in T cells, alters T cell development and function and suggest that impaired T cell activation and dysregulated cytokine production may contribute to IEI in humans.

## Results

### Constitutive disruption of Hem1 alters T cell development and results in hepatosplenomegaly.

**Figure 1:**
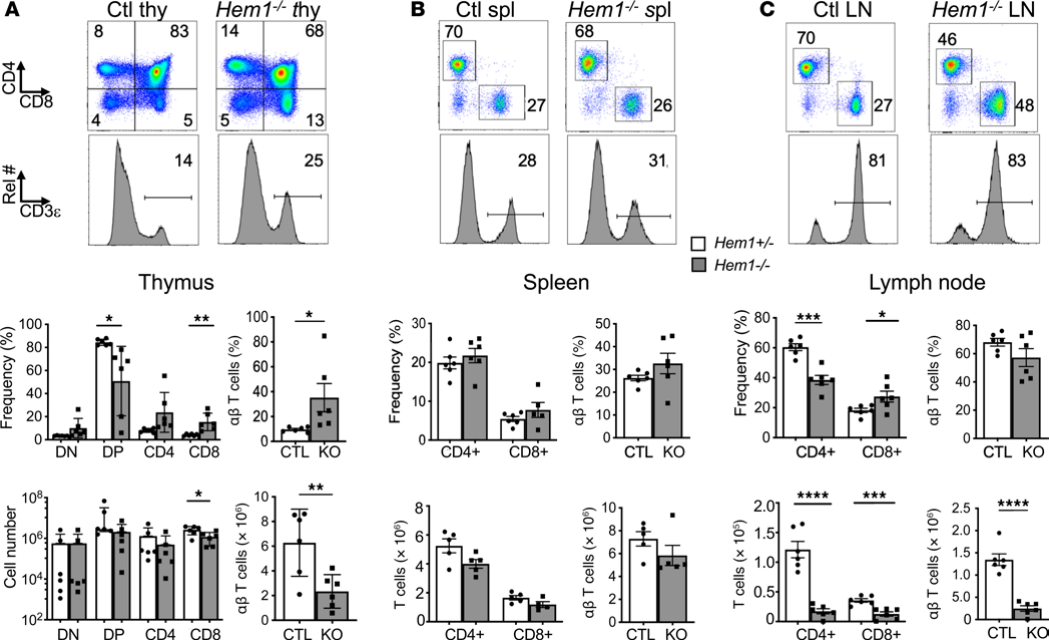
Constitutive disruption of *Hem1* disrupts T cell development. Total thymocytes, splenocytes, and cells from axillary and inguinal lymph nodes (LNs) were isolated from *Hem1^–/–^* mice and *Hem1^+/–^* littermate controls. T cell populations were analyzed by flow cytometry. (**A**) Representative flow cytometric dot plots and histograms of thymocytes. Bar graphs show quantification of double-negative (DN), double-positive (DP), CD4^+^CD8^–^ (CD4), and CD8^+^CD4^–^ (CD8) cells in the thymus. (**B**) Representative flow cytometric dot plots and histograms of splenocytes. Bar graphs show quantification of CD4^+^ and CD8^+^ T cells. (**C**) Representative flow cytometric dot plots and histograms of cells from the axillary and inguinal LNs combined. Bar graphs show quantification of CD4^+^ and CD8^+^ T cells. *n* = 6/group, 11- to 16-week-old mice; each data point represents an individual mouse. Cells were first gated on FSC/SSC lymphocytes (Supplemental Figure 1A) and then FSC-height (FSC-H) and FSC-area (FSC-A) single cells. Data are representative of 2 or more independent experiments. Data were analyzed via unpaired 2-tailed Student’s *t* test. *P < 0.05, **P < 0.01, ***P < 0.001, ****P < 0.0001.

Humans with LOF mutations in the *NCKAP1L* gene resulting in PID lack HEM1 protein in all hematopoietic tissues. To model HEM1 PID in mice, we generated constitutive *Nckap1l*-null (Hem1-null)mice using gene targeting technology (10, 12). Analyses of T cell development in thymi from 8- to 12-week-old *Hem1^–/–^* and littermate control (LMC) mice by flow cytometry revealed significant reductions in the total number of αβ thymocytes (Figure 1A and Supplemental Figure 1, A and C; supplemental material available online with this article; https://doi.org/10.1172/jci.insight.174235DS1). This correlated with slight reductions in the representation of double-positive (DP) thymocytes, with a corresponding increase in the representation of CD4^+^ and CD8^+^ single-positive (SP) T cells. Although no significant differences were noted in the representation of T cells in spleens (Figure 1B and Supplemental Figure 1, A and C), there were significant reductions in both the representation and total number of CD4^+^ αβ T cells in lymph nodes (LNs) (Figure 1C and Supplemental Figure 1C). The representation of CD8^+^ αβ T cells was increased in *Hem1^–/–^* mice, although the total numbers were decreased. In humans with Hem1 deficiency, 2 research groups reported an inversion of the CD4^+^/CD8^+^ T cell ratio (2, 4), as was noted here.

To determine effects of constitutive Hem1 loss on the development of γδ T cells, total thymocytes, splenocytes, and LN cells from *Hem1^–/–^* and LMC mice were stained with fluorescently conjugated antibodies against γδ T cell receptor (TCR) and αβ TCR, followed by flow cytometry. We found that relative to αβ T cells, the representation of γδ T cells was increased in *Hem1^–/–^* versus LMC mice in thymi, whereas the representation of γδ T cells was decreased in spleens (Supplemental Figure 2B). However, the total number of γδ T cells was decreased in all lymphoid tissues, consistent with impaired development/homeostasis of both γδ and αβ T cells following disruption of Hem1 (Supplemental Figure 2B).

In addition to the changes in T homeostasis noted here, Hem1-deficient mice presented with other gross features of Hem1-deficient humans, including increased spleen and liver weight (hepatosplenomegaly) relative to brain weight (Supplemental Figure 1D) and reduced body weight (10). Potential causes of hepatosplenomegaly in Hem1-deficient mice and patients with PID include increased extramedullary hematopoiesis, amyloidosis, and infection (9, 13).

### Increased representation of Tregs in Hem1-null mice.

**Figure 2:**
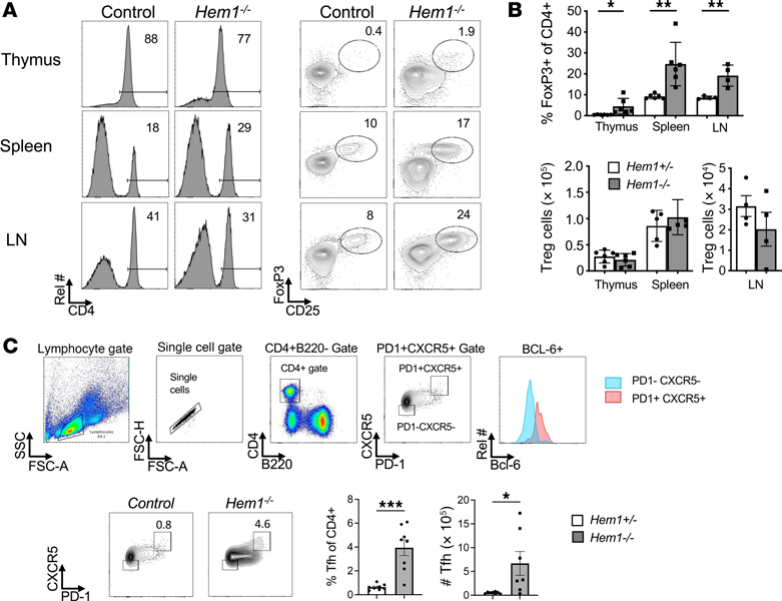
Constitutive disruption of *Hem1* results in increased proportion of Tregs and Tfh cells. (**A** and **B**) Total thymocytes, splenocytes, and cells from axillary and inguinal lymph nodes (LNs) were isolated from *Hem1^–/–^* mice and *Hem1^+/–^* littermate controls. Representative flow cytometric dot plots and histograms (**A**), and bar graphs showing quantification of Tregs (CD4^+^CD25^+^FoxP3^+^) (**B**). Cells were first gated on FSC/SSC lymphocytes and then FSC-H/FSC-A single cells. *n* = 6/group, 11- to 16-week-old mice. (**C**) Total splenocytes were isolated from *Hem1^–/–^* mice and *Hem1^+/–^* littermate controls. Representative flow cytometric dot plots and histograms with gating strategy for Tfh cells (CD4^+^CXCR5^+^PD-1^+^). Bar graphs show quantification of Tfh cells. *n* = 8/group, 11- to 14-week-old mice; each dot represents an individual mouse. Data are representative of 2–3 independent experiments. Bar graphs represent mean ± SEM and were analyzed via unpaired 2-tailed Student’s *t* test. *P < 0.05, **P < 0.01, ***P < 0.001. Treg, regulatory T cell; Tfh, T follicular helper.

To define how constitutive loss of Hem1 affects the representation of Tregs, we harvested thymi, spleens, and LNs from *Hem1^–/–^* and LMC mice and analyzed the percentage and total number of CD4^+^CD25^+^Foxp3^+^ Tregs by flow cytometry. We found that the representation of Tregs was increased in all lymphoid tissues examined, although the total numbers were equivalent to LMC mice (Figure 2, A and B). However, because total CD4^+^ and CD8^+^ αβ T cells were found to be reduced in *Hem1^–/–^* mice, these results suggest that the ratio of suppressive Tregs to effector T cells increased, which could contribute to the reduced overall immune effector functions in Hem1-null mice.

Follicular helper T cells (Tfh) are a specialized subset of CD4^+^ T cells that provide T cell help to B cells in germinal center reactions. We next examined the relative representation of Tfh cells in *Hem1^–/–^* and LMC mice. We found that the percentage and total number of CD4^+^PD1^+^CXCR5^+^Bcl6^+^ Tfh cells were increased in *Hem1^–/–^* mice (Figure 2C). These results suggest that constitutive disruption of Hem1 may have and an effect on the development and/or expansion of Tfh cells.

### Decreased naive and increased memory T cells in Hem1-null mice.

**Figure 3:**
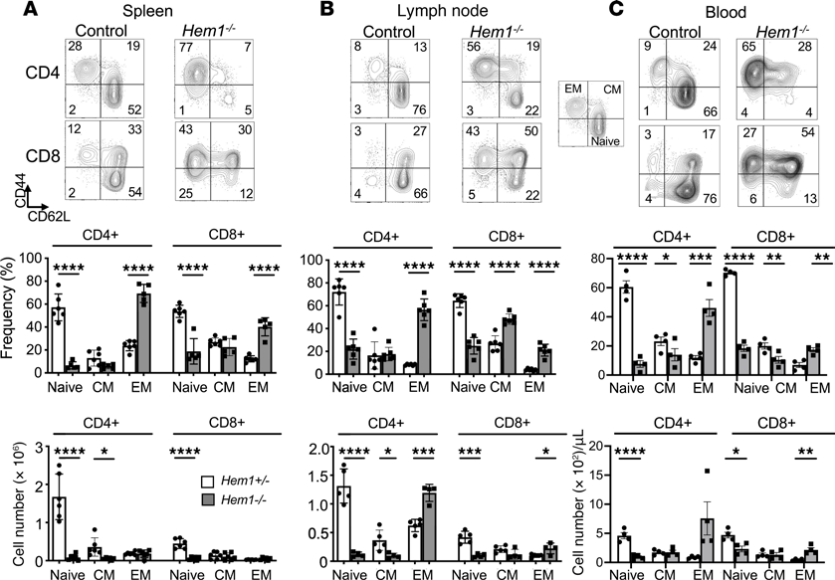
Constitutive disruption of *Hem1* results in decreased naive T cells with a concomitant increase in effector memory T cells. Total splenocytes, cells from axillary and inguinal lymph nodes, and peripheral blood were isolated from *Hem1^–/–^* mice and *Hem1^+/–^* littermate controls. (**A**) Representative flow cytometric contour plots of splenocytes. Cells were first gated on FSC/SSC lymphocytes (Supplemental Figure 1A), FSC-H/FSC-A single cells, and then either CD4^+^ or CD8^+^ cells (Figure 1, B and C). Bar graphs and quantification of naive (CD44^–^CD62L^+^), central memory (CD44^+^CD62L^+^), and effector memory (CD44^+^CD62L^–^) T cells from splenocytes. Representative flow cytometric contour plots and bar graphs of T cells harvested from (**B**) lymph nodes and (**C**) peripheral blood. *n* = 4–6/group, 11- to 16-week-old mice; each data point represents an individual mouse. Data are representative of 2 or more independent experiments. Bar graphs represent mean ± SEM and were analyzed via unpaired 2-tailed Student’s *t* test. *P < 0.05, **P < 0.01, ***P < 0.001, ****P < 0.0001. CM, central memory; EM, effector memory.

Naive T cells that have not yet encountered cognate antigen (Ag) continually recirculate between secondary lymphoid tissues and blood. After interacting with MHC-bound peptide Ag, naive T cells differentiate into effector or memory T cells that patrol nonlymphoid tissues and spleen and can reside in LNs. Upon Ag reexposure, memory T cells undergo rapid transition from a quiescent to an activated proliferative/effector state in LNs before migrating to sites of infection to help eliminate pathogens. To examine how constitutive loss of Hem1 affected the representation of naive and memory T cells, we stained splenocytes and LN cells with antibodies against CD4, CD8, CD44, and CD62L followed by flow cytometric analyses. We found that constitutive disruption of Hem1 resulted in significantly reduced percentage and total number of CD44^–^CD62L^+^ naive CD4^+^ and CD8^+^ T cells in spleen, LN, and PB, which correlated with increased percentage of CD44^+^CD62L^–^ effector memory (EM) T cells (Figure 3, A–C). The total number of CD4^+^ and CD8^+^ EM T cells was also increased in LN and PB cells (Figure 3, B and C). Decreased percentages of CD4^+^ and CD8^+^ central memory (CM) cells were noted in PB, while the total number of CD4^+^ CM cells was decreased in spleen and LN (Figure 3, A–C). These results suggest that constitutive loss of Hem1 results in a shift toward decreased naive T cells, decreased CM T cells, and increased EM T cells.

### Reduced frequency of αβ T cells and increased Tregs and memory T cells in T cell–specific Hem1-deficient mice.

**Figure 4:**
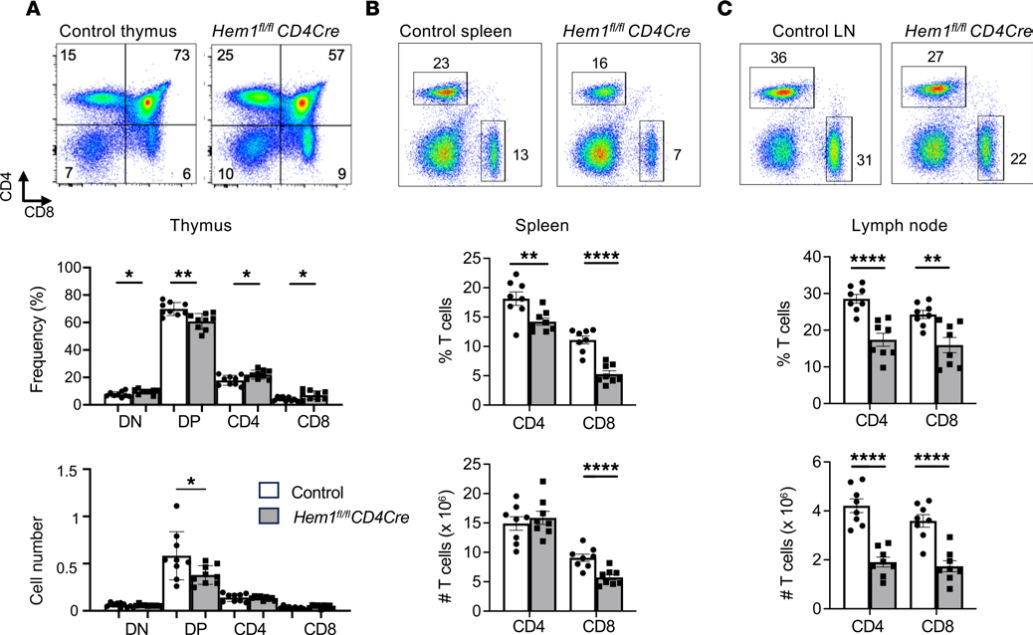
T cell–specific conditional deletion of *Hem1* disrupts T cell development. Total thymocytes, splenocytes, and lymphocytes from the axillary and inguinal lymph nodes (LNs) were isolated from *Hem1^fl/fl^CD4Cre* mice and *Hem1^fl/fl^* littermate controls. T cell populations were analyzed by flow cytometry. (**A**) Representative flow cytometric dot plots of thymocytes. Bar graphs show quantification of double-negative (DN), double-positive (DP), CD4^+^CD8^–^ (CD4), and CD8^+^CD4^–^ (CD8) cells in the thymus. (**B**) Representative flow cytometric dot plots of splenocytes. Bar graphs show quantification of CD4^+^ and CD8^+^ T cells. (**C**) Representative flow cytometric dot plot of cells from the axillary and inguinal LNs combined. Bar graphs show quantification of CD4^+^ and CD8^+^ T cells. *n* = 8–9/group, 10- to 40-week-old mice; each data point represents an individual mouse. Data are representative of 2 or more independent experiments. Cells were first gated on FSC-A/SSC-H lymphocytes and FSC-A, FSC-H single cells (Supplemental Figure 1B). Bar graphs represent mean ± SEM and were analyzed via unpaired 2-tailed Student’s *t* test. *P < 0.05, **P < 0.01, ****P < 0.0001. CTL, control; cKO, conditional knockout.

**Figure 5:**
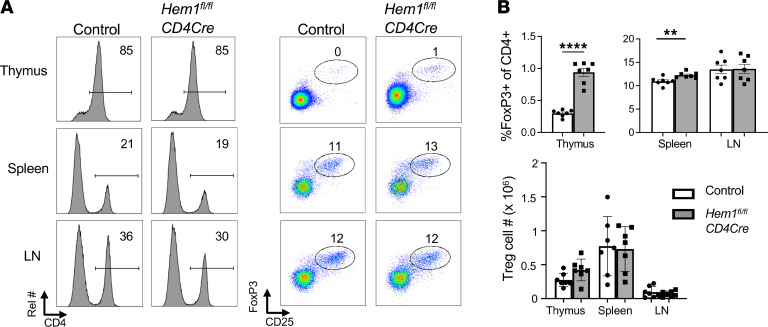
Mice with T cell–specific conditional deletion of *Hem1* have increased proportion of Tregs. (**A**) Total thymocytes, splenocytes, and cells from axillary and inguinal lymph nodes (LNs) were isolated from *Hem1^fl/fl^CD4Cre* mice and *Hem1^fl/fl^* littermate controls. Representative flow cytometric dot plots and histograms of gating strategy for Tregs. Cells were first gated on FSC/SSC lymphocytes and FSC-A/FSC-H single cells. (**B**) Bar graphs show quantification of Tregs (CD4^+^CD25^+^FoxP3^+^). *n* = 7–8/group, 10- to 12-week-old mice; each dot represents an individual mouse. Data are representative of 2 or more independent experiments. Data were analyzed via unpaired 2-tailed Student’s *t* test. **P < 0.01, ****P < 0.0001. Treg, regulatory T cell.

Having established that constitutive disruption of Hem1 mimics many aspects of T cell deficiency in Hem1 PID in humans, we next examined which of the T cell phenotypes in *Hem1^–/–^* mice are T cell autonomous. For these studies, we utilized the Cre-LoxP system where we bred *Hem1^fl/fl^* mice with mice expressing Cre under control of either the Lck proximal promoter (*pLckCre*), which deletes *lox*-flanked alleles early during the double-negative (DN) stages of T cell development (14), or *CD4Cre*, which deletes *lox*-flanked alleles during the DP stage of T cell development (15). We then isolated thymocytes, splenocytes, and LN cells and analyzed the development of αβ and γδ T cells by flow cytometry. Analyses of thymocytes from *Hem1^fl/fl^CD4Cre* versus *Hem1^fl/fl^* or *CD4Cre* mice revealed increased DN, decreased DP, and increased CD4^+^ and CD8^+^ SP thymocytes (Figure 4A), as was noted in *Hem1^–/–^* mice (Figure 1A). The representation of CD4^+^ and CD8^+^ SP T cells was decreased in the spleens and LNs of *Hem1^fl/fl^CD4Cre* mice versus LMC mice (Figure 4, B and C). The total numbers of CD4^+^ and CD8^+^ T cells were decreased in LNs, while the total number of CD8^+^ T cells was decreased in spleens of *Hem1^fl/fl^CD4Cre* versus control mice. Also as seen with *Hem1^–/–^* mice, the representation of γδ T cells was increased in thymus and LN, while the total numbers of γδ T cells were increased in LN (Supplemental Figure 3A), suggesting that disruption of Hem1 appears to have a greater effect on the homeostasis of αβ versus γδ T cells. Interestingly, the representation of CD4^+^CD25^+^Foxp3^+^ Tregs was increased in thymus and spleen, as we had seen in *Hem1^–/–^* mice (Figure 5, A and B). Hem1-deficient CD4^+^CD25^+^ Tregs suppressed anti-CD3/anti-CD28–stimulated CD4^+^ T effector cells equally well compared to control Tregs in vitro (Supplemental Figure 3B). The percentage and total number of Tfh cells were not different between *Hem1^fl/fl^pLckCre* mice versus LMC mice (data not shown) and between *Hem1^fl/fl^CD4Cre* mice versus LMC mice (Supplemental Figure 3C), suggesting that the increased representation of Tfh cells we see in *Hem1^–/–^* mice may be reflective of reductions in other hematopoietic lineage cells, and/or due to interactions with Hem1-deficient B cells. These results suggest that Hem1 regulates the development and homeostasis of CD4^+^ and CD8^+^ T cells in a T cell–specific manner.

We next assessed the representation of naive and memory T cells in spleens, LNs, and PB following T cell–specific disruption of Hem1. Similar to what was seen in *Hem1^–/–^* mice, we found decreased representation and total number of naive CD4^+^ and CD8^+^ T cells and increased representation and total number of CD4^+^ EM T cells in spleen and PB from *Hem1^fl/fl^CD4Cre* mice relative to LMC mice (Supplemental Figure 4). The percentage of CD8^+^ EM T cells was increased in spleen and LN, and the total number of CM CD8^+^ T cells was decreased in LN (Supplemental Figure 4). These results collectively suggest that Hem1 has a central role in regulating T cell memory in a T cell–specific manner.

### Decreased T cell activation and increased inhibitory receptor expression in T cell–specific Hem1-deficient mice.

**Figure 6:**
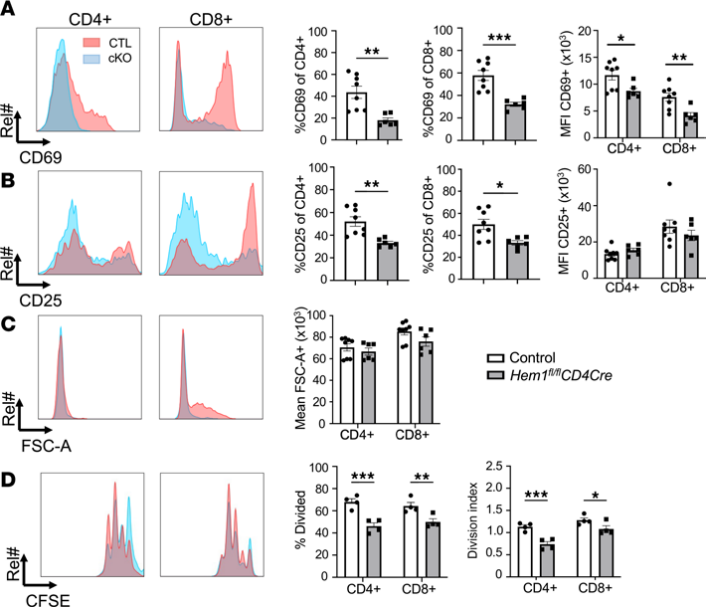
Conditional disruption of *Hem1* in T cells results in decreased CD4^+^ T cell activation and proliferation. Purified T cells from splenocytes harvested from *Hem1^fl/fl^CD4Cre* mice and *Hem1^fl/fl^* littermate controls were stimulated with anti-CD3 and anti-CD28 antibody–coated beads. Cells were first gated on lymphocytes, single cells, and then either CD4^+^ or CD8^+^ cells. CD69 and CD25 histograms and bar graphs representing the frequency and MFI of (**A**) CD69^+^ and (**B**) CD25^+^ T cells after 24 hours of stimulation. (**C**) FSC-A histogram and bar graphs representing mean FSC after 24 hours of stimulation. (**D**) Enriched T cells were stained with CFSE and then stimulated for 48 hours. CFSE proliferation histograms and bar graphs of percentage divided cells and the division index. *n* = 4–8/group, 10- to 56-week-old mice; data representative of 3 or more independent experiments, each dot represents an individual mouse. Bar graphs represent mean ± SEM and were analyzed via unpaired 2-tailed Student’s *t* test. *P < 0.05, **P < 0.01, ***P < 0.001. Rel, relative.

We had previously shown that constitutive loss of Hem1 in mice due to a noncoding point mutation (*Hem1^pt/pt^*) resulted in reduced T cell proliferation (9). To examine how T cell–specific disruption of Hem1 affected T cell activation, we measured the upregulation of the early activation markers CD69 and CD25 following 24 hours of anti-CD3 and anti-CD28 stimulation. We found that there was a significant reduction in CD69 and CD25 upregulation in CD4^+^ and CD8^+^ T cells in *Hem1^fl/fl^CD4Cre* mice versus LMC mice (Figure 6, A and B). Although we observed no significant difference in cell size (Figure 6C), using CFSE labeling we found that there was a significant reduction in the proliferation of both CD4^+^ and CD8^+^ T cells following 48 hours of anti-CD3/anti-CD28 bead stimulation (Figure 6D). These results suggest that disruption of Hem1 specifically in T cells results in a reduction in T cell activation and proliferation.

**Figure 7:**
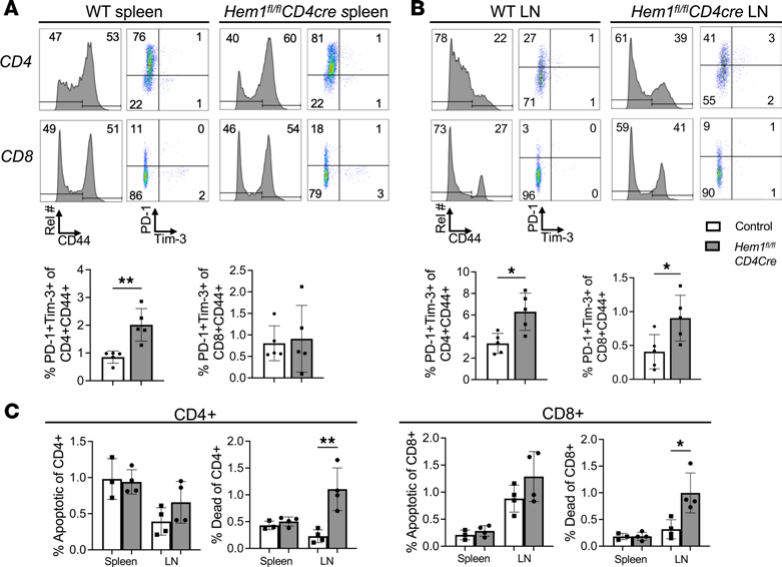
T cell–specific deletion of *Hem1* results in increased expression of exhausted T cell markers and increased cell death. Total splenocytes and cells from axillary and inguinal lymph nodes (LNs) were harvested from *Hem1^fl/fl^CD4Cre* mice and *Hem1^fl/fl^* littermate controls and analyzed by flow cytometry. (**A**) Representative gating strategy and bar graphs representing the frequency of PD-1^+^Tim-3^+^ cells of CD4^+^CD44^+^ (left) and CD8^+^CD44^+^ (right) splenocytes. (**B**) Representative flow cytometric dot plots and histograms of cells from the axillary and inguinal LNs combined. (**C**) Bar graphs represent the frequency of apoptotic (Caspase3^+^Live/Dead^–^) and dead (Caspase3^+^Live/Dead^+^) CD4^+^ (left) and CD8^+^ (right) T cells. *n* = 3–5/group, 39- to 54-week-old mice; each dot represents an individual mouse. Data representative of 2 independent experiments. Cells were first gated on FSC/SSC lymphocytes, FSC-H/FSC-A single cells, and then either CD4^+^ or CD8^+^ cells. Data were analyzed via unpaired 2-tailed Student’s *t* test. *P < 0.05, **P < 0.01.

T cells from Hem1-deficient patients were characterized as having an “exhausted” phenotype, exemplified by increased memory T cells, reduced T cell proliferation, reduced upregulation of activation markers such as CD69 and CD25, and increased inhibitory receptor expression (3, 4). Given the similarities to Hem1-deficient mice, we next evaluated the expression of the inhibitory receptors Tim3 and PD-1 on T cells from aged *Hem1^fl/fl^pLckCre* mice versus LMC mice. We found that T cell–specific disruption of Hem1 resulted in significantly increased representation of CD4^+^CD44^+^Tim3^+^PD-1^+^ and CD8^+^CD44^+^Tim3^+^PD-1^+^ splenic and LN T cells, and increased number of CD8^+^CD44^+^Tim3^+^PD-1^+^ LN T cells (Figure 7, A and B), which was associated with increased death of LN T cells (Figure 7C). These results suggest that disruption of Hem1 results in increased T cells with characteristics of exhausted cells.

### Increased TCR-induced activation of mTORC2 following T cell–specific disruption of Hem1.

**Figure 8:**
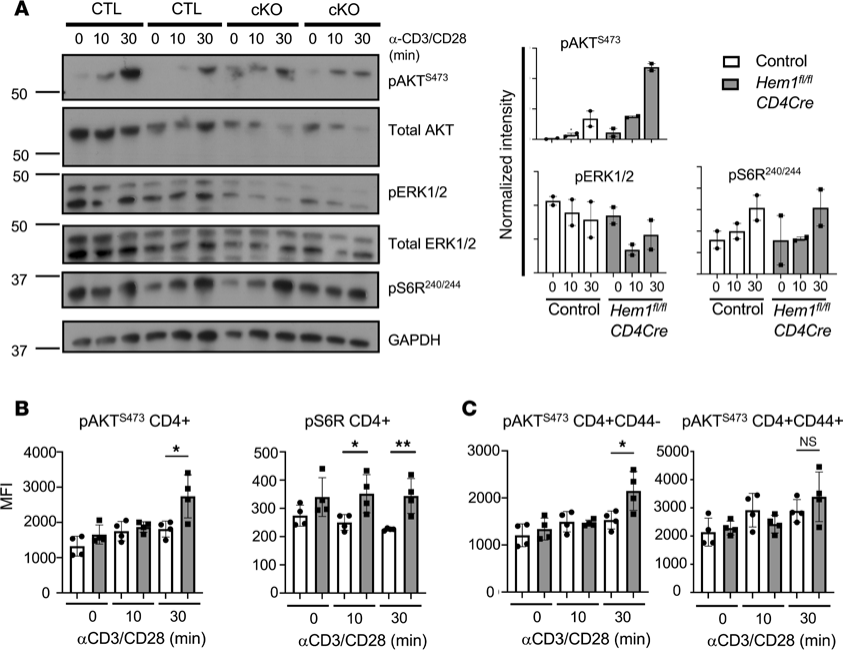
T cell–specific deletion of Hem1 results in increased mTORC signaling in CD4^+^ T cells. (A) Immunoblot of indicated proteins from lysates of purified CD4^+^ T cells from Hem1^fl/fl^CD4Cre mice and Hem1^fl/fl^ littermate controls stimulated for 0, 10, and 30 minutes with anti-CD3 and anti-CD28 antibodies. Normalized intensity was determined using ImageJ software and bar graphs represent mean ± SEM. n = 2/group, 18- to 19-week-old mice. Data representative of 3 independent experiments. **(B)** Total splenocytes harvested Hem1^fl/fl^CD4Cre mice and Hem1^fl/fl^ littermate controls and stimulated for 0, 10, and 30 minutes with anti-CD3 and anti-CD28 antibodies. Flow cytometric analysis of intracellular signaling proteins p-AKT^S473^ and p-S6R^S240/244^ levels in CD4^+^ T cells. **(C)** Flow cytometric analysis of intracellular signaling protein p-AKT^S473^ in naive (CD44^lo^) and memory (CD44^hi^) CD4^+^ T cells. Cells first gated on FSC/SSC lymphocytes, FSC-H/FSC-A single cells, and CD4^+^ T cells. See representative flow cytometric histograms in Supplemental Figure 5A. n = 4/group, 32- to 33-week-old mice. Data representative of 3 independent experiments, each dot represents an individual mouse. Data were analyzed via unpaired 2-tailed Student’s t test. *P < 0.05, **P < 0.01. NS, not significant; min, minutes; CTL, control; cKO, conditional knockout.

To examine the cellular and molecular consequences of T cell–specific loss of Hem1 during T cell activation, we measured the activation of specific cell signaling pathways by immunoblotting and intracellular flow cytometry following stimulation with anti-CD3/anti-CD28. We found that T cell–specific disruption of Hem1 in *Hem1^fl/fl^CD4Cre* mice resulted in increased mTORC2 activation based on increased levels of phosphorylated AKT at serine 473 (p-AKT^S473^) relative to total AKT via immunoblotting (Figure 8A). Analyses of Hem1-deficient T cells by intracellular flow cytometry also revealed increased mTORC2 activation based on increased p-AKT^S473^ within gated total CD4^+^ T cells and CD4^+^CD44^–^ naive T cells (Figure 8, B and C, and Supplemental Figure 5A). In addition, mTORC1 activation was noted to be increased based on increased phosphorylation of S6 ribosomal protein (p-S6R) (Figure 8B and Supplemental Figure 5A).

To examine TCR signal strength in vivo during T cell development, we utilized transgenic mice expressing GFP from the immediate early gene *Nr4a1* (*Nur77*) locus. It had previously been shown that Nur77GFP is upregulated in T cells by Ag receptor stimulation but not by inflammatory stimuli, and that GFP levels correlate with the strength of TCR stimuli (16, 17). Thus, we generated *Hem1^fl/fl^pLckCreNur77GFP* mice and *Hem1^fl/fl^Nur77GFP* control mice and assessed GFP expression during T cell development by flow cytometry. As was previously shown, we found that Nur77GFP is low in DN thymocytes and is upregulated in CD4^+^ and CD8^+^ SP cells after selection. However, we found no differences in Nur77GFP expression between Hem1-deficient and control thymocytes throughout T cell development (Supplemental Figure 5B). In addition, we found that Nur77GFP expression was equally high in peripheral CD4^+^ and CD8^+^ splenic (Supplemental Figure 5C) and LN (Supplemental Figure 5D) T cells, suggesting that there were no dramatic differences in basal TCR signaling strength during T cell development.

### Increased cytokine production, reduced cortical actin, and increased granule membrane fusion following T cell–specific disruption of Hem1.

**Figure 9:**
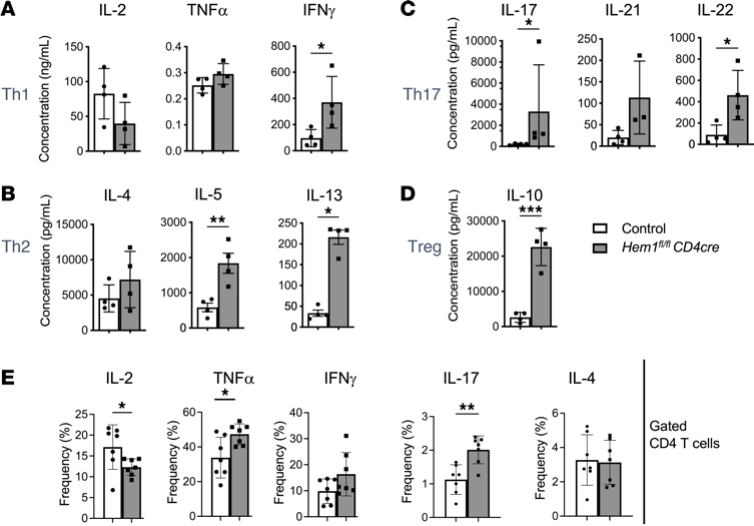
T cell–specific disruption of *Hem1* results in altered cytokine production. (**A**–**D**) Purified CD4^+^ T cells from *Hem1^fl/fl^CD4Cre* mice and *Hem1^fl/fl^* littermate controls were stimulated with anti-CD3 and anti-CD28 antibodies for 72 hours. Concentrations of cytokines in supernatant were measured by multiplex immunoassay. Cytokines produced predominantly by Th1 cells (**A**), Th2 cells (**B**), Th17 cells (**C**), and Tregs (**D**). Data representative of 1 experiment. (**E**) Purified T cells from splenocytes harvested from *Hem1^fl/fl^CD4Cre* mice and *Hem1^fl/fl^* littermate controls were stimulated with anti-CD3 and anti-CD28 antibodies for 72 hours followed by PMA and ionomycin stimulation for 5 hours. Bar graphs represent frequencies of CD4^+^ T cells expressing indicated cytokines measured by intracellular flow cytometry. Cells first gated on FSC/SSC lymphocytes, FSC-H/FSC-A single cells, and CD4^+^ T cells (Supplemental Figure 7A). Data representative of 2 or more independent experiments. *n* = 3–7/group, 11- to 15-week-old mice; each dot represents an individual mouse. Data were analyzed via unpaired 2-tailed Student’s *t* test except IL-17 in (**C**), which was analyzed via Mann-Whitney test because the data were not normally distributed. *P < 0.05, **P < 0.01, ***P < 0.001.

We next sought to determine whether T cell–specific disruption of Hem1 resulted in changes in cytokine production. We first purified total T cells from *Hem1^fl/fl^pLckCre* and LMC mice and measured cytokine release via a 31-Plex bead cytokine array 72 hours after in vitro anti-CD3/anti-CD28 stimulation. We found that loss of Hem1 resulted in decreased IL-2 and increased IL-10 and IL-17 production (Supplemental Figure 6A). To further define the changes in cytokine expression specific to CD4^+^ T cells, we purified CD4^+^ T cells from *Hem1^fl/fl^CD4Cre* and control mice and measured cytokine production via cytokine array 72 hours after anti-CD3/anti-CD28 stimulation. We found that Hem1-deficient CD4^+^ T cells produced significantly more IFN-γ (Figure 9A) and more of the Th2 cytokines IL-5 and IL-13 (Figure 9B). Similar to stimulation of total T cells, Hem1-deficient CD4^+^ T cells produced significantly more of the Th17 cytokines IL-17 and IL-22 (Figure 9C), and increased IL-10 (Figure 9D). Intracellular staining by flow cytometry confirmed decreased IL-2, increased TNF-α, and increased IL-17 (Figure 9E and Supplemental Figure 7). Supplementation of IL-2 did not rescue proliferation of Hem1-deficient T cells relative to control T cells, suggesting that IL-2 deficiency is not the only cause of reduced activation following disruption of Hem1 (Supplemental Figure 6B).

**Figure 10:**
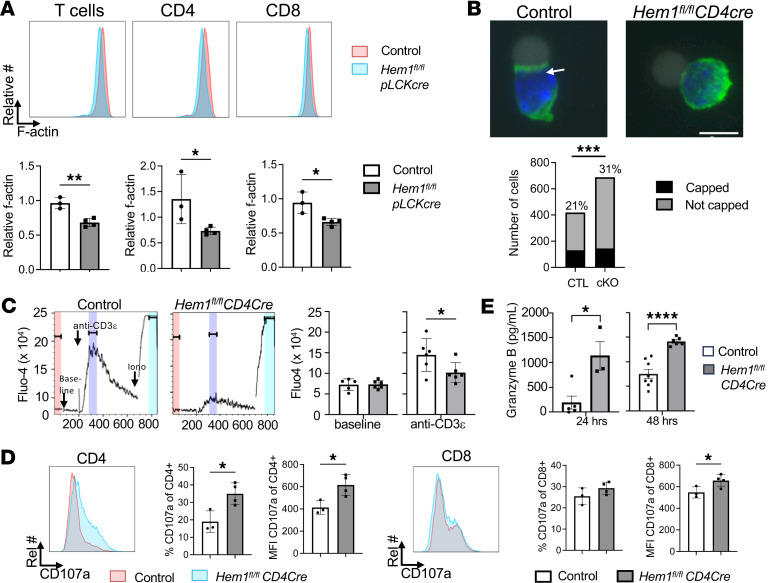
Disruption of *Hem1* results in defective F-actin polymerization, actin cap formation, and dysfunctional cortical actin leading to increased exocytosis. (**A**) Purified T cells from 10- to 14-week-old *Hem1^fl/fl^pLCKCre* mice and *Hem1^fl/fl^* littermate controls were stimulated with anti-CD3 and anti-CD28 antibodies followed by PMA and ionomycin. Representative flow cytometric histograms showing F-actin fluorescence. Cells were first gated on FSC/SSC lymphocytes, FSC-H/FSC-A single cells, T cells, CD4^+^ cells, or CD8^+^ cells. Bar graphs represent relative F-actin MFI compared to baseline. Data representative of 2 independent experiments. (**B**) Fluorescence microscopy of purified T cells stimulated with anti-CD3/anti-CD28 Dynabeads and stained for actin. Representative images captured at ×100 original magnification showing F-actin/phalloidin (green), nucleus (blue), and Dynabeads (gray). Arrow indicates actin cap. Scale bar: 5 μm. Bar graph represents capped and noncapped T cells. Percentages of capped cells are annotated. Fisher exact test using a 2 × 2 contingency table. Data representative of 1 experiment. (**C**) Impaired intracellular calcium influx. Purified T cells from *Hem1^fl/fl^CD4Cre* and *Hem1^fl/fl^* control mice were labeled with anti-CD4, anti-CD8, and the calcium binding dye Fluo-4 followed by flow cytometry. Cells were stimulated with anti-CD3ε followed by ionomycin (iono). Data are representative of 2 experiments (*n* = 6/group). (**D**) Splenocytes were harvested from 52-week-old *Hem1^fl/fl^CD4Cre* mice and *Hem1^fl/fl^* LMCs and stimulated with PMA and ionomycin for 5 hours. CD107a surface localization assessed via flow cytometry. Histograms and bar graphs represent frequency and MFI of CD4^+^ and CD8^+^ cells. Data representative of 1 experiment. (**E**) Purified T cells were stimulated with anti-CD3/anti-CD28 antibody–coated beads for 72 hours. Supernatants were harvested and granzyme B levels measured by ELISA. Shown are bar graphs depicting levels of granzyme B after stimulation. Data are representative of 2 experiments. Each dot represents an individual mouse, *n* = 3–8/group. Data were analyzed via unpaired 2-tailed Student’s *t* test unless otherwise indicated. *P < 0.05, **P < 0.01, ***P < 0.001, ****P < 0.0001.

We next investigated whether the effects of Hem1 loss on increased cytokine production could be due to changes in T cell cortical actin. It has previously been shown that a dense ring of branched actin cytoskeleton surrounds the cytosol of immune cells, which is thought to create a cortical barrier that regulates secretion of immunoregulatory vesicles (18–21). We first assessed F-actin polymerization following anti-CD3/anti-CD28 stimulation of purified T cells from *Hem1^fl/fl^pLckCre* and LMC mice via flow cytometry following staining with phalloidin, which binds actin filaments. We found that the relative MFI of phalloidin in stimulated versus unstimulated cells was decreased in CD4^+^ and CD8^+^ T cells following Hem1 disruption (Figure 10A). Analyses of TCR actin capping following stimulation of purified T cells from *Hem1^fl/fl^pLckCre* and LMC mice with anti-CD3/anti-CD28 beads revealed that Hem1 disruption resulted in a reduction in actin capping at bead interaction sites, indicative of reduced immune synapse formation (Figure 10B). This correlated with decreased calcium influx following stimulation with anti-CD3ε, suggesting that decreased actin capping has important functional consequences (Figure 10C). We next assessed levels of T cell surface CD107a, which measures granule-membrane fusion and exocytosis, in *Hem1^fl/fl^CD4Cre* and control mice. We found that loss of Hem1 resulted in significantly increased CD107a expression on both CD4^+^ and CD8^+^ T cells, consistent with increased membrane fusion following disruption of Hem1 (Figure 10D).

Decreased cortical actin and increased CD107a has been associated with increased granule-membrane fusion in cytotoxic T lymphocytes (CTLs) (22–25). To determine whether disruption of Hem1 has an effect on granzyme release, we measured levels of granzyme B in supernatant via ELISA following TCR stimulation of purified *Hem1^fl/fl^CD4Cre* T cells relative to *Hem1^fl/fl^* T cells. We found that disruption of Hem1 resulted in increased levels of granzyme B in supernatant 24 and 48 hours after stimulation (Figure 10E). These results suggest that Hem1-deficient T cells have a hypersecretory cytokine and granzyme phenotype and reduced IL-2, which is associated with reduced actin capping, reduced calcium influx, and increased membrane granule fusion and exocytosis.

### T cell–specific disruption of Hem1 does not result in increased autoantibody formation.

Increased autoantibody production is one feature of humans with LOF mutations in Hem1 (3), and increased autoantibodies were noted in constitutive Hem1-null mice (3) and B cell–specific Hem1-deficient mice (10). Given the changes in T cell signaling and cytokine release in our murine models, we next assessed whether T cell–specific disruption of Hem1 was sufficient to result in increased autoantibody production. Sera were collected from aged female *Hem1^fl/fl^pLckCre* mice and *Hem1^fl/fl^* control mice (49–56 weeks old), and IgM and IgG autoantibody production was assessed using autoantigen microarray technology containing 128 autoantigens. Only one significant increase in IgG (PM/Scl-100, a protein component of the PM/Scl RNA processing complex, *P* < 0.04) and IgM (SmD, a small nuclear riboprotein involved in mRNA splicing, *P* < 0.04) autoantibodies were noted in serum from *Hem1^fl/fl^pLckCre* versus control mice (Supplemental Figure 8), whereas 33 IgM and 34 IgG autoantibodies were elevated in serum from mice following B cell–specific disruption of Hem1 (10). These results suggest that loss of Hem1 only in T cells is not sufficient to result in increased autoantibodies in Hem1-deficient mice.

## Discussion

Defining the cellular and molecular functions of gene variants resulting in IEIs can be challenging in humans due to the limited number of patients, genetic heterogeneity, concurrent infections, and autoimmunity and hyperinflammatory syndromes that result in treatment with immunosuppressive agents. In this study, we utilized constitutive and conditional gene targeting technologies in mice to investigate the T cell–autonomous roles of Hem1 in regulating T cell development and functions. Our results suggest that Hem1 has critical roles in regulating the development, homeostasis, and effector functions of T cells, in part by controlling T cell signaling and the actin cytoskeleton. Importantly, our results also reveal considerable conservation in phenotypes between Hem1-deficient humans and mice (see Supplemental Tables 1 and 2), thus supporting the utilization of cell-type-specific gene targeting in mice to dissect the importance of Hem1 in overall immunity.

Both constitutive and T cell–specific loss of Hem1 resulted in reductions in the representation of CD4^+^ or CD8^+^ αβ T cells in spleen and/or LNs. The reduction in αβ T cells correlated with an increase in the representation of γδ T cells in constitutive and T cell–specific Hem1-deficient mice. A reduction in the representation of CD4^+^ T cells and an increase in representation of γδ T cells was also noted in human patients with PID (3, 4). Although there were no significant differences in the representation and number of Tfh cells following T cell–specific disruption of Hem1, the representation of CD4^+^CD25^+^Foxp3^+^ Tregs was significantly increased following both constitutive and T cell–specific disruption of Hem1. This correlated with an approximately 7-fold increase in IL-10 production following anti-CD3/anti-CD28 stimulation of purified CD4^+^ T cells in vitro, which may be derived from CD4^+^Foxp3^–^ Tr1 cells and/or CD4^+^Foxp3^+^ conventional Tregs. These results suggest that an increase in the ratio of immunosuppressive Tregs versus T effector cells following disruption of Hem1 may contribute to altered immune function in Hem1-deficient mice and humans.

In addition to a reduction in the representation of αβ T cells, both constitutive and T cell–specific disruption of Hem1 resulted in a reduction in naive CD4^+^ and CD8^+^ T cells and increased EM CD4^+^ and CD8^+^ splenic T cells. T cell–specific Hem1-null mice also expressed increased levels of T cell exhaustion markers, including PD-1 and Tim3 on CD44-gated memory T cells. Decreased naive CD4^+^ and CD8^+^ T cells and increased EM T cells were also a consistent feature of Hem1-deficient human patients with PID (2–4), and 2 studies found increased exhaustion and senescence markers in patient CD4^+^ and CD8^+^ T cells (3, 4). These results suggest that both Hem1-deficient humans and mice exhibit a shift toward increased memory T cells with features of T cell exhaustion, perhaps secondary to impaired naive T cell output and prolonged immune stimulation associated with impaired pathogen clearance, autoimmunity, and/or cytokine-driven hyperinflammation.

Given the reduction in peripheral T cells and exhaustion-like phenotype, we assessed the effects of Hem1 loss on T cell activation, proliferation, and survival. We found that both CD4^+^ and CD8^+^ T cells from T cell–specific Hem1-deficient mice had reduced upregulation of the early activation markers CD69 and CD25. In addition, Hem1-deficient CD4^+^ and CD8^+^ cells divided less efficiently than control cells 48 hours after stimulation. Previous studies have shown that Hem1-deficient human T cells have reduced CD69 and CD25 upregulation (2–4) and reduced proliferative potential of CD4^+^ T cells (2) or total T cells (3). Analyses of human T cells disrupted for Hem1 revealed decreased phosphorylation of AKT at serine 473 following TCR/CD28 stimulation, indicative of decreased mTORC2 activation, whereas phosphorylation of S6R indicative of mTORC1 signaling proceeded normally (2, 3). Cook et al. found that Hem1 coimmunoprecipitated with Rictor, a component of the mTORC2 complex, suggesting that Hem1 may have a role outside of the WAVE complex in regulating mTORC2 enzymatic activity. In contrast, we found that disruption of Hem1 resulted in increased mTORC2 activation via immunoblotting and intracellular flow cytometry, which was consistent with another report that T cell–specific disruption of murine WAVE2 resulted in increased mTORC1 and mTORC2 activation (26). These results suggest that human and mouse T cells may respond slightly differently to loss of the WAVE complex at the molecular level.

Controlled polymerization and depolymerization of the actin cytoskeleton are important for multiple active processes in immune cells, including formation of the immunological synapse and granule release. For example, a dense ring of cortical actin lies beneath the cell surface membrane, which likely serves as a barrier to prevent excessive secretion of vesicles and their cargo such as granzymes and cytokines (see ref. 27 for a review). Given the importance of Hem1 in actin regulation, we assessed how T cell–specific disruption of Hem1 altered cytokine and granzyme release following TCR/CD28 stimulation. We found that loss of Hem1 only in T cells resulted in significantly increased release of the Th1 cytokine IFN-γ, Th2 cytokines IL-5 and IL-13, Th17 cytokines IL-17 and IL-22, and the Treg cytokine IL-10 from purified CD4^+^ T cells following anti-CD3/anti-CD28 stimulation. Interestingly, only IL-2 production appeared to decrease in Hem1-deficient T cells, most likely due to a reduction in intracellular calcium signaling downstream of TCR stimulation. Actin polymerization and immune synapse formation was reduced following T cell–specific disruption of Hem1, and expression of surface CD107a, a marker of exocytosis-based granule release, was significantly higher in both CD4^+^ and CD8^+^ T cells. This also correlated with increased release of granzyme B following TCR/CD28 stimulation. Although IL-17 was not noted to be increased in any of the human Hem1 PID studies, serum IFN-γ and IL-10 levels were noted to be increased by Castro et al., and IL-2 production was noted to be decreased by CD4^+^ cells following TCR/CD28/ICAM1 stimulation by Cook et al. In addition, increased levels of CD107a were noted in both studies (2, 4), and release of granzymes A and B was found to be increased in one study (2). These results are consistent with the notion that disruption of Hem1 specifically in T cells results in impaired immune synapse formation and a reduction in the cortical actin barrier, which may contribute to vesicle hypersecretion and cytokine and granzyme release following TCR/CD28 stimulation.

The selective decrease in IL-2 production found in Hem1-deficient mice and humans is most likely due to reduced actin capping at the immune synapse, as was noted in Hem1-deficient mouse and human T cells. Multiple studies have concluded that the integrity of the actin cytoskeleton is necessary to establish and maintain prolonged contacts between T cells and APCs, in part by dynamically maintaining the shape and microarchitecture of the T cell immune synapse (see refs. 8, 28 for review). F-actin–dependent events at the immune synapse include control of synapse assembly, shape, and polarity; formation of TCR and coreceptor microclusters; organelle trafficking/positioning; anchoring of the centrosome to the nucleus and cell polarization; and endosomal recycling. Although TCR proximal signaling often appears minimally perturbed, disruption of actin polymerization either through gene mutations (i.e., *WASp*, *WAVE2*) or pharmacologically (i.e., cytochalasin) consistently impairs *IL-2* transcription, in part by inhibiting calcium signaling pathways and NFAT nuclear import (29–31). Thus, our findings provide evidence that Hem1 is important for a linear signaling pathway initiated by efficient F-actin polymerization, immune synapse formation, and intracellular calcium signaling leading to optimal IL-2 production.

The consistent increases in IL-17 production we observed following constitutive and T cell–specific disruption of Hem1 is particularly noteworthy, given the associations of Th17 cells and disease in humans. For example, Cook et al. found that 5 out of 5 Hem1-deficient children from 4 independent families presented with asthma symptoms (2). IL-17 has been proposed to play a major role in Th2-low asthma (32–35). Higher levels of IL-17 are found in serum, sputum, and bronchoalveolar lavage fluid of patients with asthma (36–40). IL-17 promotes inflammation in part by stimulating granulopoiesis, neutrophil recruitment/activation (41, 42), and fibrosis (43, 44). Several studies also showed that concurrent increased expression of IL-17 and IL-22 in mononuclear cells and bronchial biopsies correlated with more severe asthma that was resistant to steroids (37, 45–47). Blockade of IL-17 using anti–IL-17 antibodies or gene targeting in murine models of asthma reduced mucus hypersecretion, goblet cell hyperplasia, subepithelial collagen deposition, airway smooth muscle thickening, and airway neutrophilia (48–51). These results collectively suggest that loss of Hem1 specifically in T cells may contribute to asthma in Hem1-deficient children in part by increasing production of IL-17 and IL-22. In a recent systemic review, increased serum IL-17 was also consistently noted in humans with food allergies, allergic rhinitis, and atopic dermatitis, and higher IL-17 correlated with the severity of disease (52). Thus, increased IL-17 may also contribute to increased incidence of allergic diseases in Hem1-deficient children.

Another important link between loss of Hem1, increased IL-17, and human disease is the association of Hem1 and inflammatory bowel disease (IBD). Using GWAS, Peters et al. identified *NCKAP1L* as 1 of 12 key driver genes in human IBD (53), and increased frequencies of IL-17A^+^ cells were found in intestinal lamina propria of *Nckap1l^pt/pt^* mice deficient in Hem1. Th17 cells have been shown to be potent mediators of IBD in part through production of IL-17 and IFN-γ, and by stimulating myoblasts to secret matrix metalloproteinases (MMPs), which penetrate multiple components of the extracellular matrix leading to epithelial cell damage (54, 55; see ref. 56 for review). IL-17 also promotes recruitment of inflammatory cells by stimulating epithelial cells to secrete chemokines such as IL-8. Our results suggest that production of IL-17 by CD4^+^ T cells may be a key mediator of IBD in humans, and that loss of Hem1 specifically in CD4^+^ T cells may contribute to intestinal disease in Hem1-deficient children by secreting IL-17.

Overall, studies on Hem1 deficiency using murine models provide important opportunities to dissect cellular and molecular functions of Hem1 using methods that might not be possible using human samples. Importantly, studies on mice deficient in Hem1 in all tissues (*Hem1^pt/pt^*, *Hem1^–/–^*) show significant similarities to humans deficient in Hem1. Clinically, Hem1-null mice are severely immunodeficient, presenting with recurrent infections, hepatosplenomegaly, atopic disease, autoimmune disease, and failure to thrive, similar to human patients with PID (3, 9, 12). Histological analyses of Hem1-null mice revealed increased inflammation in multiple organs, including lung, heart, liver, pancreas, large intestine, epididymis, kidney, with amyloid deposition at liver margins (3, 6, 9, 53), features also noted in human patients with PID (5). Utilization of cell-type-specific conditional gene targeting using the Cre-LoxP system provided opportunities to further dissect the cell-autonomous consequences of Hem1 loss on specific immune cells. For example, disruption of Hem1 specifically in myeloid cells confirmed the cell-autonomous importance of Hem1 in neutrophil migration and phagocytosis. In addition, a previously unrecognized role for Hem1 in the development of alveolar macrophages and limiting proinflammatory cytokine release was also uncovered (12), perhaps revealing an important contribution of Hem1 loss in lung immunity and disease. Conditional disruption of Hem1 specifically in B cells highlighted B cell–specific roles for Hem1 in limiting B cell hyperactivation, production of age-associated B cells, IFN-γ production, autoantibody production, and providing protection against *Streptococcus pneumoniae* challenge (10). In the current study, we extend these finding by demonstrating the T cell–specific importance of Hem1 in driving optimal T cell activation, proliferation, and homeostasis, and maintaining properly regulated cytokine and granzyme release, likely through maintenance of the immune synapse and cortical actin barrier. Our results suggest that increased IL-17 and Th2 cytokine production by Hem1-deficient CD4^+^ T cells may contribute to dermal atopy and asthma, and potentially IBD in Hem1-deficient mice and children. Similarly, an increased ratio of Tregs to effector T cells and altered CD4^+^ and CD8^+^ T cell effector functions could contribute to immunodeficiency disease. Finally, disruption of Hem1 specifically in T cells was not sufficient to result in increased autoantibody production, suggesting that the combined effects of Hem1 loss in multiple immune cell types contribute to massive immune dysregulation, consisting of immunodeficiency, hyperinflammation, and autoimmunity.

## Methods

Further information can be found in Supplemental Methods.

### Sex as a biological variable.

These studies included both male and female mice, and sex was not considered as a biological variable.

### Mice.

*Hem1^fl/fl^* mice (12) were bred with mice expressing Cre recombinase under the control of proximal promoter of the lymphocyte protein tyrosine kinase (*pLckCre*) [B6.Cg-Tg(Lck-cre)548Jxm/J] (14) to generate mice with T cell–specific deletion of Hem1 early in thymocyte development at the DN stage 2. *Hem1^fl/fl^* mice were also bred with mice expressing Cre recombinase under the under control of the *CD4* enhancer/promoter/silencer sequence (*CD4Cre*) [B6.Cg-Tg(Cd4-cre)1Cwi/BfluJ] (15) to generate mice with T cell–specific deletion of Hem1 later in thymocyte development at the CD4^+^CD8^+^ DP stage. Mice with constitutive deletion of Hem1 (*Hem1^–/–^*) were generated as previously described (12).

*Hem1^fl/fl^pLckCre* and *Hem1^fl/fl^CD4Cre* mice were screened and maintained by genomic PCR analysis as described for *pLckCre* (14) and *CD4Cre* (15) and following amplification with *Hem1^fl/fl^* forward and reverse oligonucleotides (12). Mice were housed under specific pathogen–free conditions. Experiments were performed on mice from generation 6–10 on a C57BL/6J background. No phenotypic differences were noted between male and female mice; thus, both sexes were used in the experiments. Most studies were performed on mice ages 8–20 weeks, with the exception of some experiments performed on mice aged more than 20 weeks. Autoantibody and exhausted T cell marker experiments were performed in aged mice between the ages of 38 and 54 weeks. LMCs were utilized whenever possible and in most experiments. Experimental controls included both *Hem1^+/–^* and *Hem1^fl/fl^* mice when studying the constitutive and conditional knockout models, respectively.

### Flow cytometry.

Murine thymocytes, splenocytes, and LN cells were harvested and splenocytes underwent RBC lysis using ammonium chloride potassium (ACK) lysis buffer (Invitrogen/Life Technologies) prior to staining. Cells were stained with fluorescent dye–conjugated antibodies from (i) BioLegend: CD3 (145-2C11), CD4 (GK1.5), CD8a (53-6.7), TCR β chain (H57-597), TCR (GL3), CD69 (H1.22F3), CXCR5 (L138D7), B220 (RA3-6B2), NK1.1 (PK136), PD-1 (29F.1A12), Tim-3 (RMT3-23), IL-2 (JES6-5H4), IL-17A (TC11-18H10.1) TNF-α (MP6-XT22), IFN-γ (XMG1.2), CD107a (1D4B); (ii) Tonbo Bioscience: CD25 (7D4), CD62L (MEL-14); (iii) eBioscience: p-AKT (S473) (SDRNR), p-Erk1/2 (E10), (FJK-16spS6(S235/S236) (cupk43k); (iv) Invitrogen: FoxP3(12-5773-82), IL-4 (11B11) and caspase 3/7 (C10427); (v) BD Biosciences: Bcl-6 (K112-91); and (vi) BD Pharmigen: CD44 (IM7). The Vybrant CFDA SE Cell Tracer Kit, LIVE/DEAD Fixable Near-IR Dead Cell Stain Kit, and Fluo-4 were from Invitrogen.

Intracellular staining was performed using the Fixation/Permeabilization kit (BD Biosciences), and intranuclear staining was performed using the Foxp3/Transcription Factor Staining Buffer Set (eBioscience) per manufacturer guidelines. Phosphoprotein staining was performed by fixing cells with paraformaldehyde and permeabilizing with 100% ice-cold methanol. Flow cytometric data were acquired on FACSCanto II or Symphony A2 or A3 cytometers (BD Biosciences), and FlowJo software (BD Biosciences) was used for data analysis.

### T cell stimulation ex vivo.

Splenocytes were RBC lysed as described above. T cells were enriched by magnetic bead purification using the Pan T Cell Isolation Kit II, CD4 (L3T4) MicroBeads, or Naive CD4^+^ T Cell Isolation Kit (Miltenyi Biotec). Purified T cells (2 × 10^5^) were plated in each well of a 96-well plate and stimulated with 10 mg/mL Ultra-LEAF purified anti-CD3 (17A2 or 145-2C11, BioLegend) and 10–12 mg/mL anti-CD28 antibodies (37.51, BioLegend) in complete RPMI 1640 media (RPMI plus L-glutamine, 10% FBS, 1% penicillin streptomycin, 1% MEM amino acids, 1% sodium pyruvate, 0.1% 2-mercaptoethanol) for indicated lengths of time.

### Cell proliferation assay.

Splenocytes were enriched for T cells and incubated with 5 mM CFDA using the Vybrant CFDA SE Cell Tracer Kit. Stained cells were stimulated as described above for 48 and 72 hours using either plate bound anti-CD3/anti-CD28 or Dynabeads Mouse T-Activator CD3/CD28 (Thermo Fisher Scientific) at a 1:1 bead-to-cell ratio in B cell growth media.

### Cytokine multiplex assay.

Splenocytes were enriched for T cells or CD4^+^ T cells as described above and stimulated with 10–25 mg/mL anti-CD3 and anti-CD28 antibody–coated plates for 72 hours. Supernatant was collected and analyzed using the Mouse Cytokine Array/Chemokine Array 31-Plex and the Mouse Cytokine Th17 12-Plex Discovery Assay Array (Eve Technologies). IL-2, TNF-α, and IFN-γ were reanalyzed at a 1:50 dilution in PBS following oversaturation at full concentration using the Mouse Cytokine Proinflammatory Focused 10-Plex Discovery Assay Array (Eve Technologies).

### Intracellular cytokine assay.

Purified T cells were stimulated as described above for 72 hours, followed by incubation with 50 ng/mL PMA (Sigma-Aldrich), 1 mg/mL ionomycin (Sigma-Aldrich), and brefeldin A (BioLegend) in complete RPMI 1640 for 5 hours. Cells were permeabilized with Fixation/Permeabilization kit (BD Biosciences) and stained for analysis by flow cytometry.

### Granzyme B assay.

T cells were isolated from splenocytes and activated using Dynabeads Mouse T-Activator CD3/CD28 (Thermo Fisher Scientific) at a 1:1 bead-to-cell ratio for 48 hours in B cell growth media. The supernatant was collected to measure granzyme B release using the Mouse Granzyme B DuoSet ELISA kit (R&D Systems). Cells were stained with CD4 and CD8a antibodies, followed by intracellular staining for granzyme B (NGZB, Thermo Fisher Scientific), and analyzed by flow cytometry.

### CD107a assay.

The method was adapted from Betts et al. (57), with modifications. Splenocytes were harvested and 1 × 10^6^ cells were stimulated with 50 ng/mL PMA and 1 mg/mL ionomycin in complete RPMI 1640 media for 4 hours. Anti-CD107a antibody (ID4B, BioLegend) was added at the start of stimulation, and monensin (BioLegend) was added after 1 hour of stimulation. Cells were stained and analyzed by flow cytometry.

### Calcium flux assay.

Intracellular calcium signaling was performed according to manufacturer’s instructions. Cells were stained with Fluo-4 for 30 minutes at 37°C, followed by surface staining with CD4-APC and CD8-PB. Cells were run on a FACSymphonyA2 (BD Biosciences). Baseline flux was assessed for 1 minute, followed by 10 mg biotinylated anti-CD3. Two minutes later, 20 mg streptavidin was added followed by 1 mg ionomycin 8 minutes later.

### F-actin phalloidin assay.

The F-actin assay was described previously (9). Purified T cells were stimulated as described above for 24 hours followed by stimulation with 50 ng/mL PMA (Sigma-Aldrich) and 1 mg/mL ionomycin (Sigma-Aldrich) for 15 minutes. Cells were permeabilized and stained with Alexa Fluor 488 phalloidin (ActinGreen, Invitrogen) per manufacturer guidelines and analyzed by flow cytometry.

### Fluorescence microscopy.

Purified T cells were stimulated with Mouse T-Activator Dynabeads (Thermo Fisher Scientific) at a 1:1 dilution for 15 minutes. Cells were stained with phalloidin (see above) and Hoechst 33342 (NucBlue, Invitrogen) and mounted on slides using cytospin. Cells imaged by fluorescence microscopy (Nikon Eclipse 50i and Visitech VT-iSIM).

### Immunoblots.

Purified CD4^+^ T cells were stimulated for 0, 15, and 30 minutes as described above, and lysates prepared in RIPA buffer (Invitrogen) with Halt phosphatase and protease inhibitors (Invitrogen). Protein quantification of lysates was performed using the DC Protein Assay kit (Bio-Rad). Immunoblot assays were run as previously described (10) using rabbit polyclonal antibodies specific for GAPDH (D16H11), p-AKT^S473^ (D9E), AKT (C67E7), p-S6R^240/244^ (D68F8), p-Erk1/2 (D13.14.4E), and Erk1/2 (137F5) followed by anti-rabbit IgG HRP (Promega). Densitometry analysis was performed using ImageJ software (NIH).

### Statistics.

Data were analyzed using the Student’s 2-tailed unpaired *t* test with equal variance using GraphPad Prism 9. A *P* value of less than 0.05 was considered significant. Normality was assessed using the Shapiro-Wilk normality test, and nonparametric data were analyzed using the Mann-Whitney test using GraphPad Prism. For comparison of categorical data, Fisher’s exact test was used for statistical analysis of a 2 × 2 contingency table using GraphPad Prism. Bar graphs represent mean ± SD unless otherwise specified in the figure legends.

### Study approval.

All studies involving animals were approved by the University of Washington Animal Care and Use Committee.

### Data availability.

Data are available from the corresponding author upon request. Values for all data points in graphs are reported in the Supporting Data Values file.

## Author contributions

AC, JTT, NS, and BMI designed the experiments and wrote the manuscript. AC, BMI, NS, RC, JTT, JYT, and FT collected and analyzed data. CZ generated and analyzed autoantibody array data.

## Supplementary Material

Supplemental data

Unedited blot and gel images

Supporting data values

## Version 1 07/08/2025

In-Press Preview

## Version 2 08/22/2025

Electronic publication

## References

[1] Tangye SG (2022). Human inborn errors of immunity: 2022 update on the classification from the International Union of Immunological Societies Expert committee. J Clin Immunol.

[2] Cook SA (2020). HEM1 deficiency disrupts mTORC2 and F-actin control in inherited immunodysregulatory disease. Science.

[3] Salzer E (2020). The cytoskeletal regulator HEM1 governs B cell development and prevents autoimmunity. Sci Immunol.

[4] Castro CN (2020). NCKAP1L defects lead to a novel syndrome combining immunodeficiency, lymphoproliferation, and hyperinflammation. J Exp Med.

[5] Cook S (2022). HEM1 actin immunodysregulatory disorder: genotypes, phenotypes, and future directions. J Clin Immunol.

[6] Christodoulou A (2024). Hem1 inborn errors of immunity: waving goodbye to coordinated immunity in mice and humans. Front Immunol.

[7] Park H (2010). Hem-1: putting the “WAVE” into actin polymerization during an immune response. FEBS Lett.

[8] Dupre L (2021). Actin dynamics at the T cell synapse as revealed by immune-related actinopathies. Front Cell Dev Biol.

[9] Park H (2008). A point mutation in the murine Hem1 gene reveals an essential role for Hematopoietic protein 1 in lymphopoiesis and innate immunity. J Exp Med.

[10] Avalos A (2022). Hem-1 regulates protective humoral immunity and limits autoantibody production in a B cell-specific manner. JCI Insight.

[11] Chan MM (2013). Hematopoietic protein-1 regulates the actin membrane skeleton and membrane stability in murine erythrocytes. PLoS One.

[12] Suwankitwat N (2021). The actin-regulatory protein Hem-1 is essential for alveolar macrophage development. J Exp Med.

[13] Shao L (2018). The Wave2 scaffold Hem-1 is required for transition of fetal liver hematopoiesis to bone marrow. Nat Commun.

[14] Hennet T (1995). T-cell-specific deletion of a polypeptide N-acetylgalactosaminyl-transferase gene by site-directed recombination. Proc Natl Acad Sci U S A.

[15] Lee PP (2001). A critical role for Dnmt1 and DNA methylation in T cell development, function, and survival. Immunity.

[16] Moran AE (2011). T cell receptor signal strength in Treg and iNKT cell development demonstrated by a novel fluorescent reporter mouse. J Exp Med.

[17] Zikherman J (2012). Endogenous antigen tunes the responsiveness of naive B cells but not T cells. Nature.

[18] Gil-Krzewska A (2018). An actin cytoskeletal barrier inhibits lytic granule release from natural killer cells in patients with Chediak-Higashi syndrome. J Allergy Clin Immunol.

[19] Porat-Shliom N (2013). Multiple roles for the actin cytoskeleton during regulated exocytosis. Cell Mol Life Sci.

[20] Wollman R, Meyer T (2012). Coordinated oscillations in cortical actin and Ca^2+^ correlate with cycles of vesicle secretion. Nat Cell Biol.

[21] Meunier FA, Gutierrez LM (2016). Captivating new roles of F-actin cortex in exocytosis and bulk endocytosis in neurosecretory cells. Trends Neurosci.

[22] Ritter AT (2017). Cortical actin recovery at the immunological synapse leads to termination of lytic granule secretion in cytotoxic T lymphocytes. Proc Natl Acad Sci U S A.

[23] Betts MR, Koup RA (2004). Detection of T-cell degranulation: CD107a and b. Methods Cell Biol.

[24] Gomez-Lomeli P (2014). Increase of IFN-γ and TNF-α production in CD107a + NK-92 cells co-cultured with cervical cancer cell lines pre-treated with the HO-1 inhibitor. Cancer Cell Int.

[25] Krzewski K (2013). LAMP1/CD107a is required for efficient perforin delivery to lytic granules and NK-cell cytotoxicity. Blood.

[26] Liu M (2021). WAVE2 suppresses mTOR activation to maintain T cell homeostasis and prevent autoimmunity. Science.

[27] Dustin ML, Choudhuri K (2016). Signaling and polarized communication across the T cell immunological synapse. Annu Rev Cell Dev Biol.

[28] Cassioli C, Baldari CT (2022). Lymphocyte polarization during immune synapse assembly: centrosomal actin joins the game. Front Immunol.

[29] Holsinger LJ (1998). Defects in actin-cap formation in Vav-deficient mice implicate an actin requirement for lymphocyte signal transduction. Curr Biol.

[30] Nolz JC (2006). The WAVE2 complex regulates actin cytoskeletal reorganization and CRAC-mediated calcium entry during T cell activation. Curr Biol.

[31] Zhang J (1999). Antigen receptor-induced activation and cytoskeletal rearrangement are impaired in Wiskott-Aldrich syndrome protein-deficient lymphocytes. J Exp Med.

[32] Habib N (2022). Current understanding of asthma pathogenesis and biomarkers. Cells.

[33] Margelidon-Cozzolino V (2022). Role of Th17 cytokines in airway remodeling in asthma and therapy perspectives. Front Allergy.

[34] Linden A, Dahlen B (2014). Interleukin-17 cytokine signalling in patients with asthma. Eur Respir J.

[35] Sorbello V (2015). Nasal IL-17F is related to bronchial IL-17F/neutrophilia and exacerbations in stable atopic severe asthma. Allergy.

[36] Bullens DM (2006). IL-17 mRNA in sputum of asthmatic patients: linking T cell driven inflammation and granulocytic influx?. Respir Res.

[37] Ricciardolo FLM (2017). Identification of IL-17F/frequent exacerbator endotype in asthma. J Allergy Clin Immunol.

[38] Al-Ramli W (2009). T(H)17-associated cytokines (IL-17A and IL-17F) in severe asthma. J Allergy Clin Immunol.

[39] Agache I (2010). Increased serum IL-17 is an independent risk factor for severe asthma. Respir Med.

[40] Bullone M (2019). Elevated serum IgE, oral corticosteroid dependence and IL-17/22 expression in highly neutrophilic asthma. Eur Respir J.

[41] Hellings PW (2003). Interleukin-17 orchestrates the granulocyte influx into airways after allergen inhalation in a mouse model of allergic asthma. Am J Respir Cell Mol Biol.

[42] Wilson RH (2009). Allergic sensitization through the airway primes Th17-dependent neutrophilia and airway hyperresponsiveness. Am J Respir Crit Care Med.

[43] Bellini A (2012). Interleukin (IL)-4, IL-13, and IL-17A differentially affect the profibrotic and proinflammatory functions of fibrocytes from asthmatic patients. Mucosal Immunol.

[44] Hayashi H (2013). IL-17A/F modulates fibrocyte functions in cooperation with CD40-mediated signaling. Inflammation.

[45] Zhu J (2011). Increased expression of aryl hydrocarbon receptor and interleukin 22 in patients with allergic asthma. Asian Pac J Allergy Immunol.

[46] Badi YE (2022). Mapping atopic dermatitis and anti-IL-22 response signatures to type 2-low severe neutrophilic asthma. J Allergy Clin Immunol.

[47] Wang B (2017). Increased expression of Th17 cytokines and interleukin-22 correlates with disease activity in pristane-induced arthritis in rats. PLoS One.

[48] Zhao J (2013). Th17 responses in chronic allergic airway inflammation abrogate regulatory T-cell-mediated tolerance and contribute to airway remodeling. Mucosal Immunol.

[49] Camargo LDN (2017). Effects of anti-IL-17 on inflammation, remodeling, and oxidative stress in an experimental model of asthma exacerbated by LPS. Front Immunol.

[50] Fukuzaki S (2021). Preventive and therapeutic effect of anti-IL-17 in an experimental model of elastase-induced lung injury in C57Bl6 mice. Am J Physiol Cell Physiol.

[51] Camargo LDN (2020). Bronchial vascular remodeling is attenuated by anti-IL-17 in asthmatic responses exacerbated by LPS. Front Pharmacol.

[52] Hofmann MA (2021). Role of IL-17 in atopy-A systematic review. Clin Transl Allergy.

[53] Peters LA (2017). A functional genomics predictive network model identifies regulators of inflammatory bowel disease. Nat Genet.

[54] Raza A, Shata MT (2012). Letter: pathogenicity of Th17 cells may differ in ulcerative colitis compared with Crohn’s disease. Aliment Pharmacol Ther.

[55] Lee JY (2020). Serum amyloid A proteins induce pathogenic Th17 cells and promote inflammatory disease. Cell.

[56] Jiang P (2023). The involvement of TH17 cells in the pathogenesis of IBD. Cytokine Growth Factor Rev.

[57] Betts MR (2003). Sensitive and viable identification of antigen-specific CD8+ T cells by a flow cytometric assay for degranulation. J Immunol Methods.

