# Supplementary material for: Hem1 controls T cell activation, memory, and the regulated release of immunosuppressive and proinflammatory cytokines
Source: JCI Insight. 2025 Jul 8;10(16):e174235. doi: 10.1172/jci.insight.174235 (PMC12406723; doi:10.1172/jci.insight.174235)
Supplement: Supplemental data [file jciinsight-10-174235-s123.pdf]

Supplemental Materials and Methods

Protein array profiling analysis.

Mouse sera were collected, aliquoted, and stored at -80°C. Autoantigen microarrays were manufactured in the Microarray and Immune Phenotyping Core Facility of University of Texas Southwestern Medical Center and analyzed as previously described (1).

Cell proliferation assay with IL-2 rescue

Cells were incubated with or without the addition of 100 ng/ml IL-2 (R&D Systems, Inc) to the stimulation. All other steps follow the previously described cell proliferation assay.

Treg Suppression Assay

Splenocytes were isolated and RBC lysed as described previously. $CD4^{+}CD25^{+}$ Treg cells and $CD^{+}CD25^{-}$ T effector (Teff) cells were enriched by magnetic bead purification using the $CD4^{+}CD25^{+}$ Regulatory T Cell Isolation Kit (Miltenyi Biotec). Purified Teff cells were incubated with 5mM of CFDA Vybrant™ CFDA SE Cell Tracer Kit (Thermo Fischer Scientific, Maltham, MA). Treg cells and labeled Teff cells were plated in a 96 well plate at 0:1, 1:1, and 2:1 dilutions and stimulated with Mouse T-Activator Dynabeads (Thermo) at a 1:5 bead:cell ratio for 72 hours. Teff proliferation was measured using flow cytometry.

Mice

Nur77-GFP reporter mice ($Nur77^{GFP}$) (B6N.B6-Tg(Nr4a1-EGFP/cre)820Khog/J) were supplied by the Zikherman laboratory (Zikherman *et. al*, 2012; Moran *et al.,* 2011). $Hem1^{fl/fl}pLCKcre$

mice were bred to *Nur77*^{GFP} mice to create a reporter mouse where TCR signaling induces expression of GFP as a reporter for TCR signal strength (Moran *et al.,* 2011).

Supplemental Figures

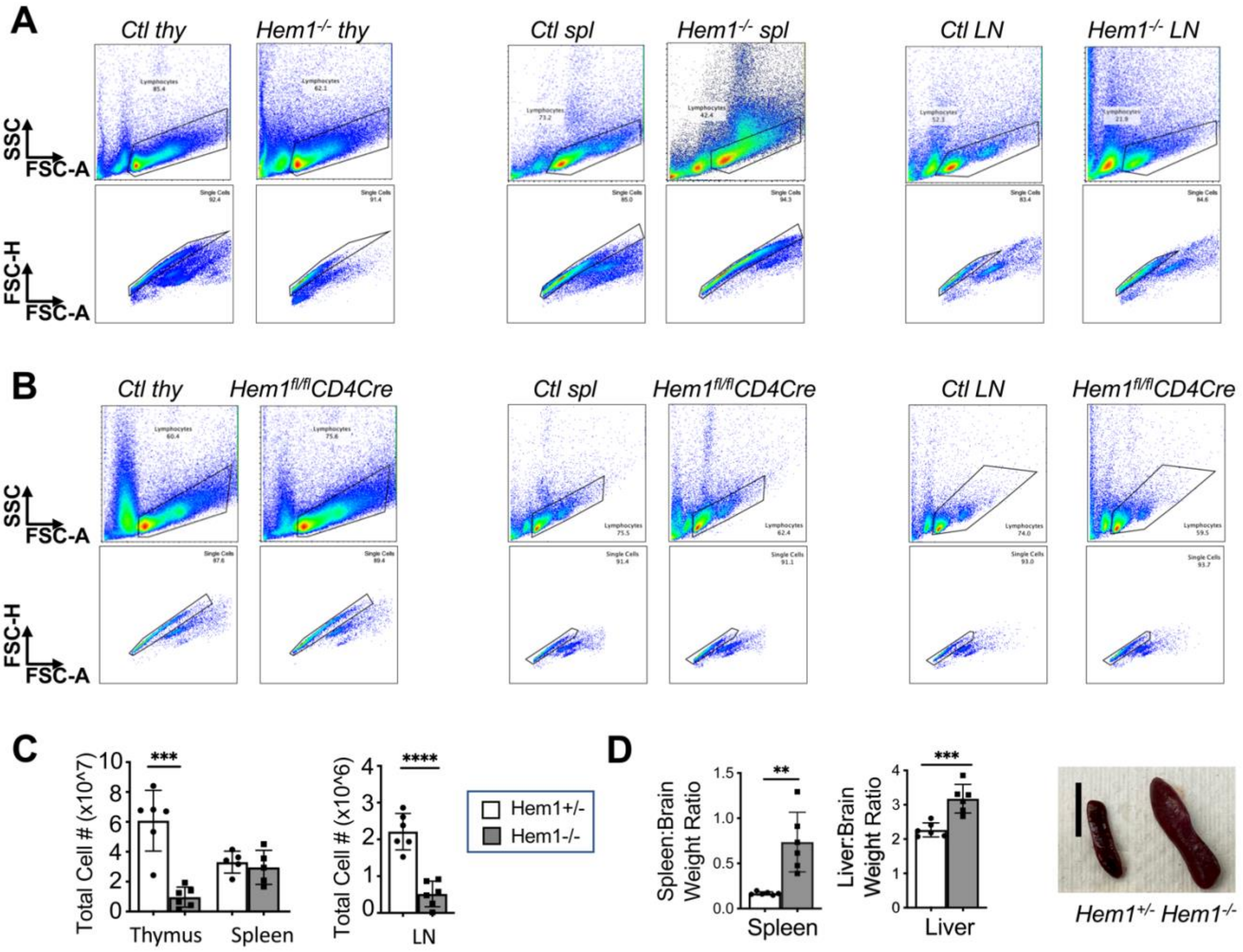

Supplemental Figure 1. Constitutive disruption of *Hem1* results in decreased cellularity in the thymus and peripheral lymph nodes and hepatosplenomegaly.

Total thymocytes, splenocytes, and cells from the axillary and inguinal lymph nodes were collected. **(A)** Representative flow cytometric dot plots showing lymphocyte gating strategy on Side Light Scatter (SSC) versus Forward Light Scatter (FSC-A) and single cell gating on FSC Height (FSC-H) versus FSC-A for *Hem1*$^{-/-}$ mice and *Hem*$^{+/-}$ littermate controls. **(B)** Representative flow cytometric dot plots for *Hem1*$^{fl/fl}$*CD4Cre* mice and *Hem1*$^{fl/fl}$ littermate controls. **(C)** Bar graphs represent the total cellularity in each organ specified harvested from *Hem1*$^{-/-}$ mice and *Hem*$^{+/-}$ littermate controls. Cell counts were calculated using Countbright absolute counting beads by flow cytometry. **(D)** Representative image of spleens harvested from *Hem*$^{+/-}$ control (left) and *Hem1*$^{-/-}$ (right) mice. Scale bar represents 1cm. 11–16-week-old mice, n=6/group, each data point represents an individual mouse. Bar graphs represent mean ± SD and were analyzed via unpaired Student's t test. **$P < 0.01$, ***$P < 0.001$, ****$P < 0.0001$. Ctl = control; Thy = thymus; Spl = spleen; LN = lymph node

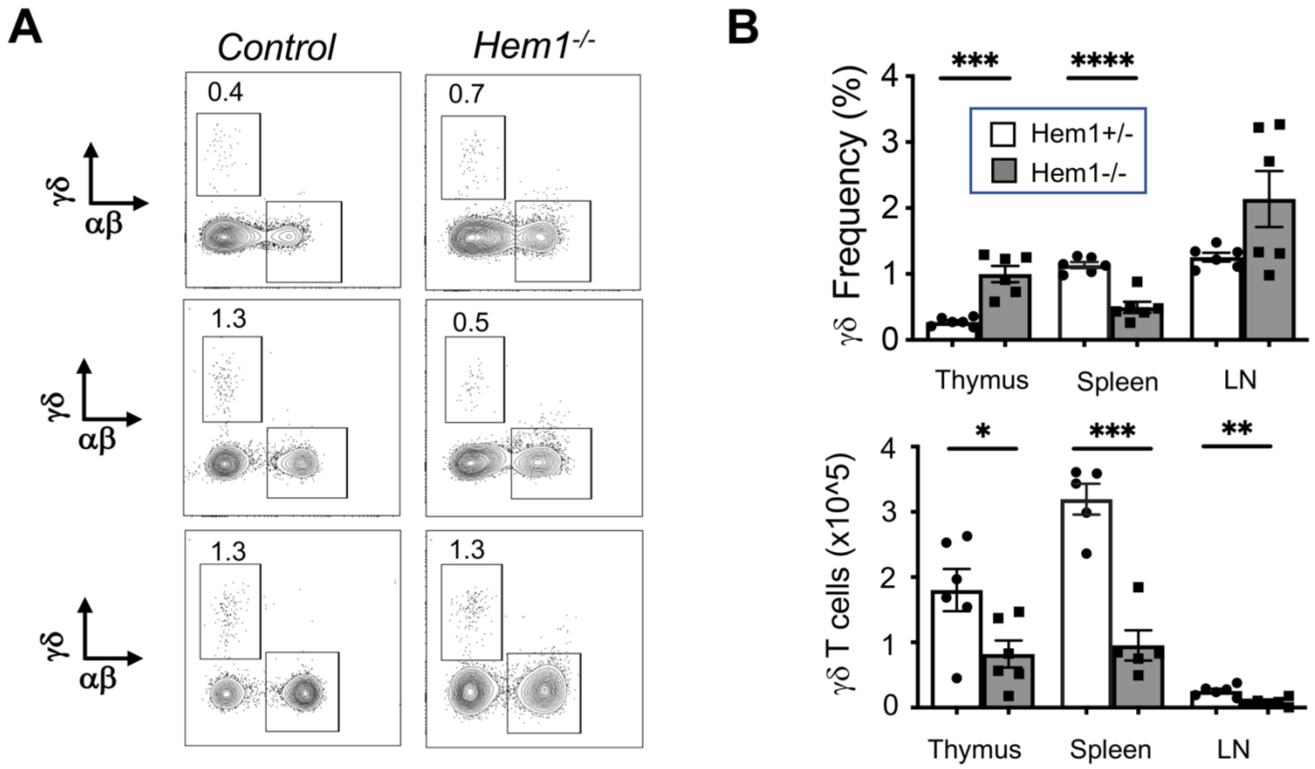

Supplemental Figure 2. Constitutive disruption of *Hem1* results in decreased numbers of γδ T cells.

Total thymocytes, splenocytes, and cells from the axillary and inguinal lymph nodes were collected from $Hem1^{-/-}$ mice and $Hem^{+/-}$ littermate controls and analyzed by flow cytometry. **(A)** Representative contour plots for γδ and αβ T cells. Cells were first gated on lymphocytes and single cells. **(B)** Bar graphs and quantification of γδ T cells. 11-16-week-old mice, n=6/group, each data point represents an individual mouse. Bar graphs represent mean ± SD and were analyzed via unpaired Student's t test. *$P < 0.05$, **$P < 0.01$, ***$P < 0.001$, ****$P < 0.0001$.

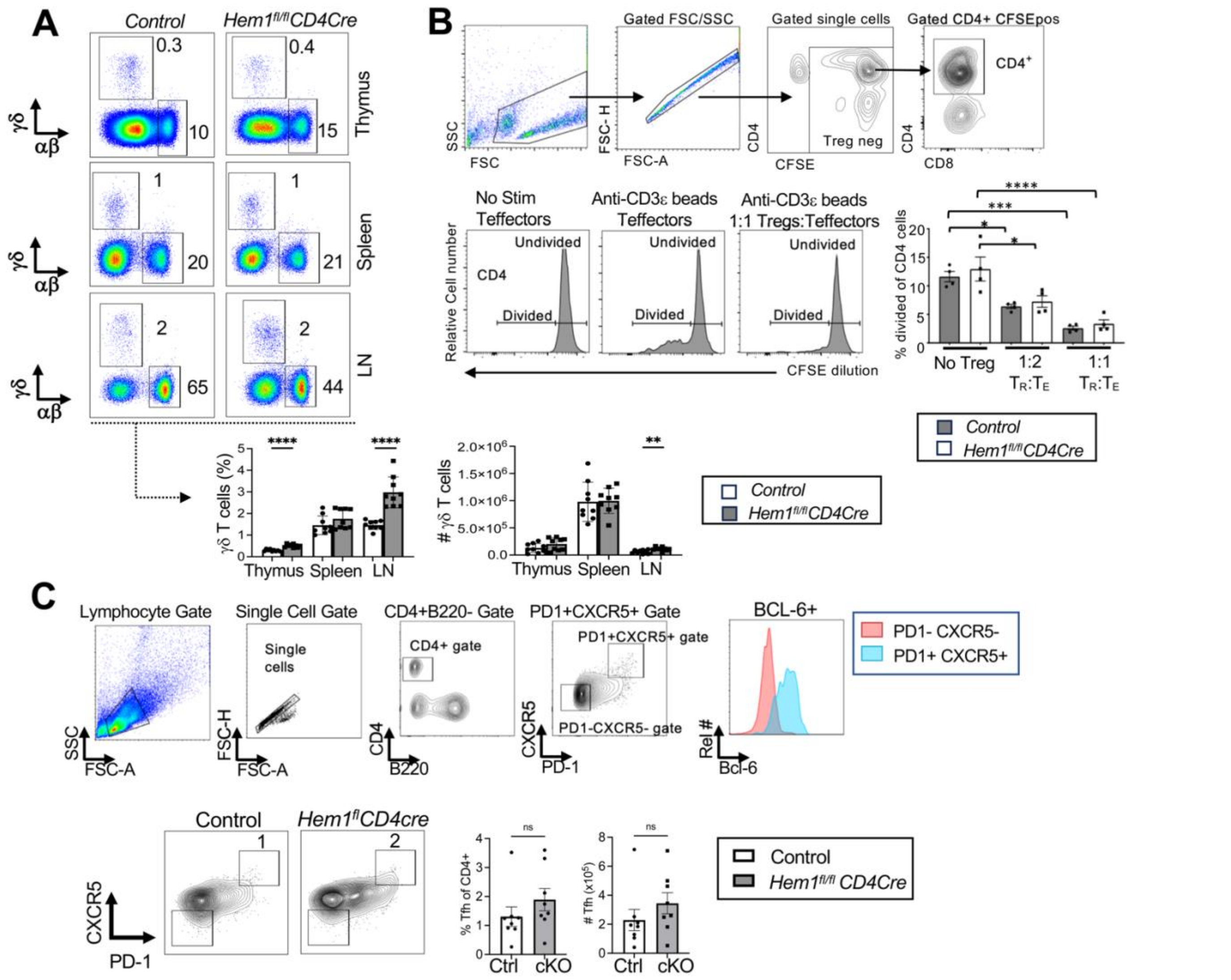

Supplemental Figure 3. T cell-specific deletion of *Hem1* results in increased frequency of γδ T cells, functional regulatory T cells, and normal frequency of T Folllicular Helper cells. Total thymocytes, splenocytes, and cells from the axillary and inguinal LNs were harvested from *Hem1$^{fl/fl}$CD4Cre* mice and *Hem1$^{fl/fl}$* littermate controls and analyzed by flow cytometry. (A) Representative flow cytometric dot plots for γδ and αβ T cells. Cells were first gated on lymphocytes. Bar graphs show quantification of γδ T cells. 10-12-week-old mice, n=6-9/group. **(B)** Purified CD4^{+}CD25^{+} Tregs (T_R) from *Hem1$^{fl/fl}$CD4Cre* or *Hem1$^{fl/fl}$* control mice were mixed with CFSE labeled CD4^{+}CD25^{-} T effectors (T_E) from *Hem1$^{fl/fl}$* mice at ratios of either no Tregs, 1:1 T_R:T_E, or 1:2 T_R:T_E. T_E were stimulated with CD3/CD28 coated beads for 72 hrs and then harvested and stained for flow cytometric analyses. (*top*) Representative flow cytometric dot plots and histograms with gating strategy to look at cell division of CD4^{+}CD25^{-} T cells. (*bottom*) Percentage of divided CD4 T_E cells in the presence or absence of Tregs. **(C)** Representative flow cytometric dot plots and histograms with gating strategy for T follicular helper cells. Bar graphs show quantification of Tfh cells (CD4^{+}CXCR5^{+}PD-1^{+}). 10-40-week-old mice, n=8/group. Each data point represents an individual mouse. Bar graphs represent mean ± SEM and were analyzed via ANOVA with multiple comparisons (B), or unpaired Student's t test (A and C). **P* < 0.05, ****P* < 0.001, *****P* < 0.0001. T_R = T regulatory; T_E = T effector; Tfh = T follicular helper; Rel = relative; CTL = control; cKO = conditional knockout.

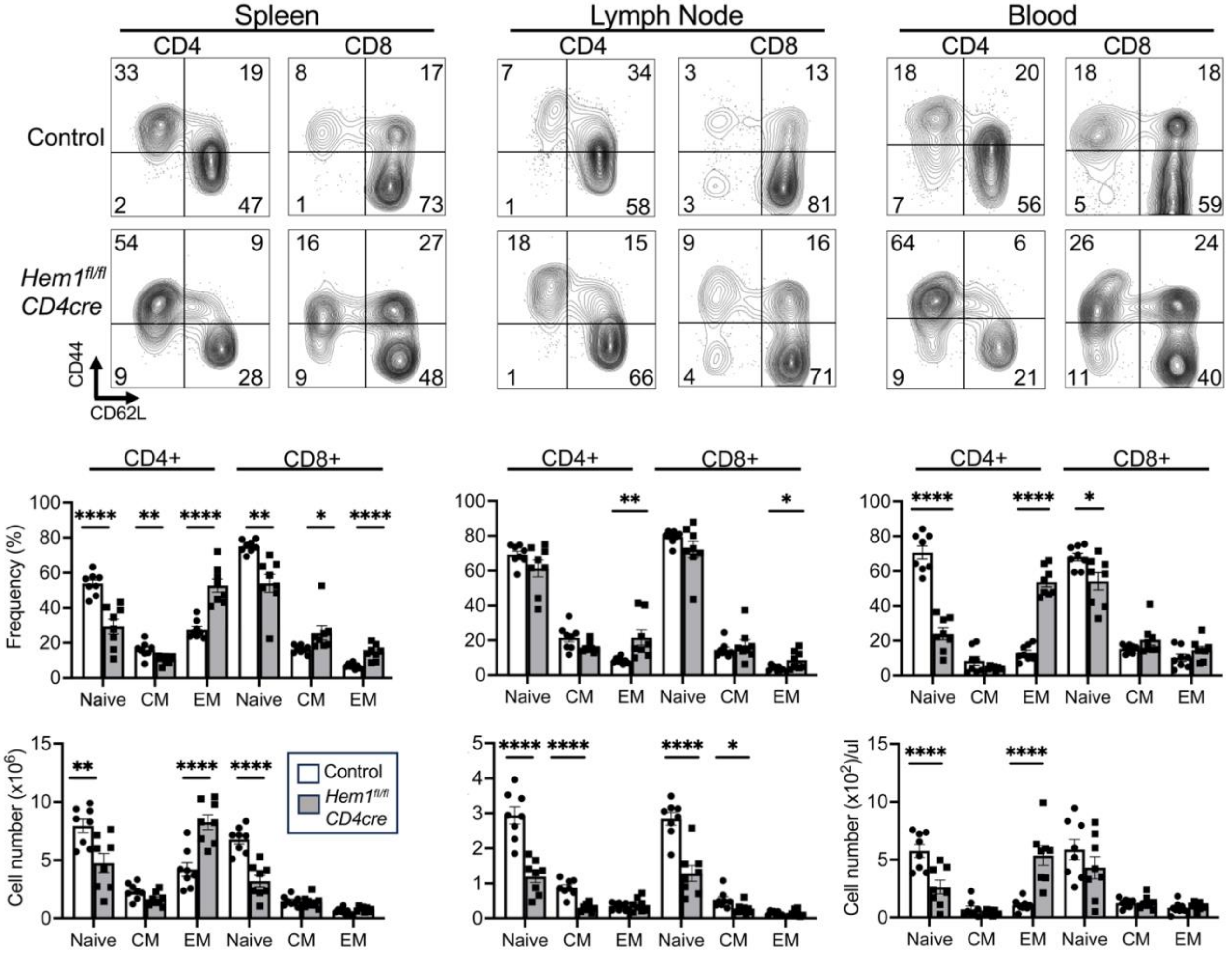

Supplemental Figure 4. T cell-specific disruption of *Hem1* results in decreased frequency of naïve T cells with concurrent increase in effector memory T cells.

Total splenocytes and cells from axillary and inguinal lymph nodes were isolated from *Hem1$^{fl/fl}$CD4Cre* mice and *Hem1$^{fl/fl}$* littermate controls. **(A)** Representative flow cytometric dot plots of splenocytes. Cells were first gated on lymphocytes, and then CD4$^+$ and CD8$^+$ cells (Figure 4B and C). Bar graphs and quantification of naïve (CD44$^-$CD62L$^+$), central memory (CD44$^+$CD62L$^+$), and effector memory (CD44$^+$CD62L$^-$) T cells from splenocytes. **(B)** Representative contour plots and bar graphs of T cells harvested from lymph nodes, and peripheral blood **(C)**. 10–40-week-old mice, n=7-8/group, each data point represents an individual mouse. Data are representative of 2 or more independent experiments. Bar graphs represent mean ± SEM and were analyzed via unpaired Student's t test. *P < 0.05, **P < 0.01, ***P < 0.001, ****P < 0.0001. CM = central memory; EM = effector memory.CM = central memory.

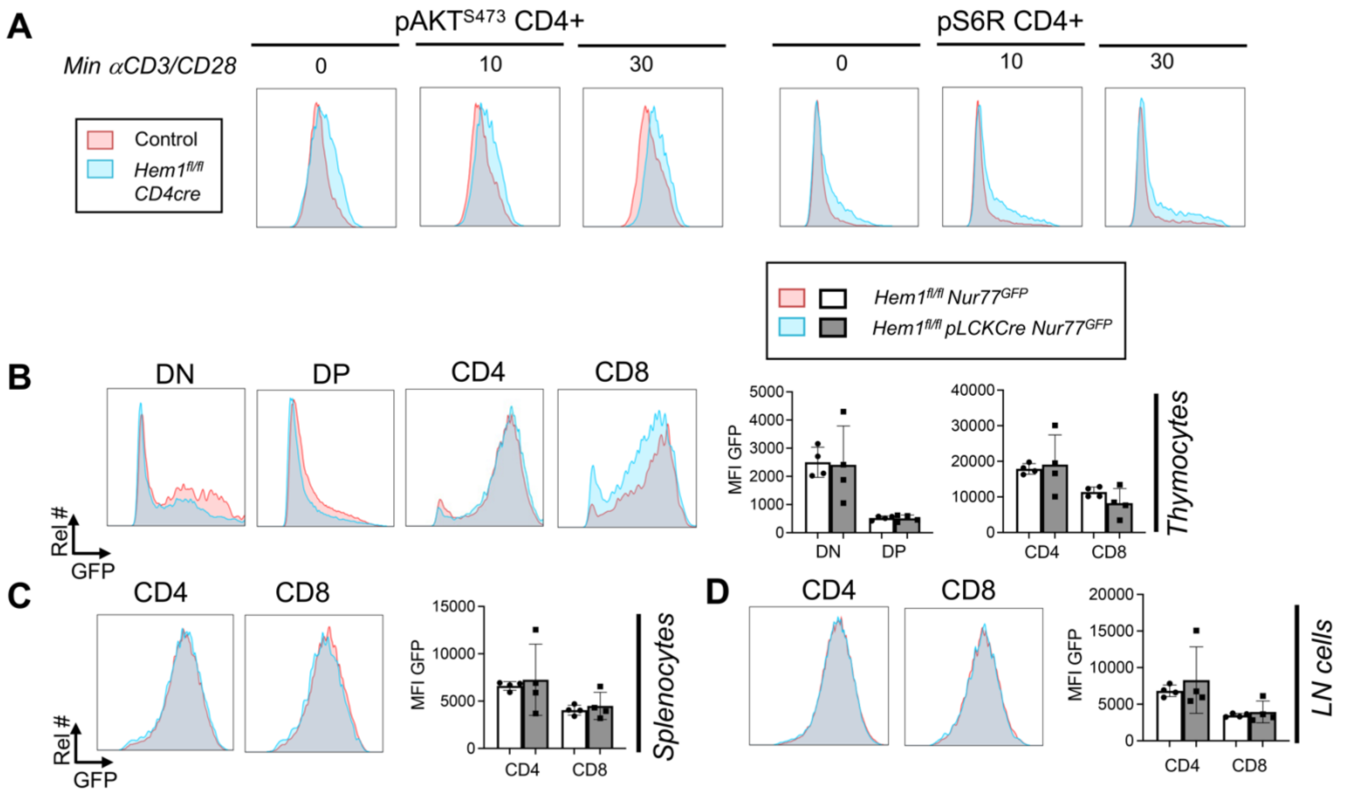

Supplemental Figure 5. T cell-specific deletion of *Hem1* results in increased mTOR signaling in CD4⁺ T cells but no observed change in upstream TCR signal strength.
(A) Representative flow cytometric histograms of phospho-AKT(Ser^{473}) and phospho-S6R(Ser$^{235/236}$) levels in CD4$^+$ T cells over 0, 10, and 30 minutes of stimulation with anti-CD3/anti-CD28 antibodies (Figure 8). Cells first gated on FSC/SSC lymphocytes, FSC-H/FSC-A single cells, and CD4$^+$ T cells. 32-33-week-old mice, n=4/group. **(B-D)** Total thymocytes, splenocytes, and cells from axillary and inguinal lymph nodes were collected from *Hem1$^{fl/fl}$pLCKCreNur77^{GFP}* mice and *Hem1$^{fl/fl}$Nur77^{GFP}* littermate controls. Representative flow cytometric histograms of GFP expression and bar graphs of MFI of GFP. Cells first gated on lymphocytes and single cells. Thymocyte expression of GFP gated on double negative (DN), double positive (DP), CD4$^+$CD8$^-$ (CD4), and CD8$^+$CD4$^-$ (CD8) cells in the thymus (B). Expression of GFP gated on CD4$^+$ and CD8$^+$ T cells in the spleen (C) and peripheral lymph nodes (D). 9-14-week-old mice, n= 4/group, each data point represents an individual mouse. Bar graphs represent mean ± SD and were analyzed via unpaired Student's t test. Rel = relative.

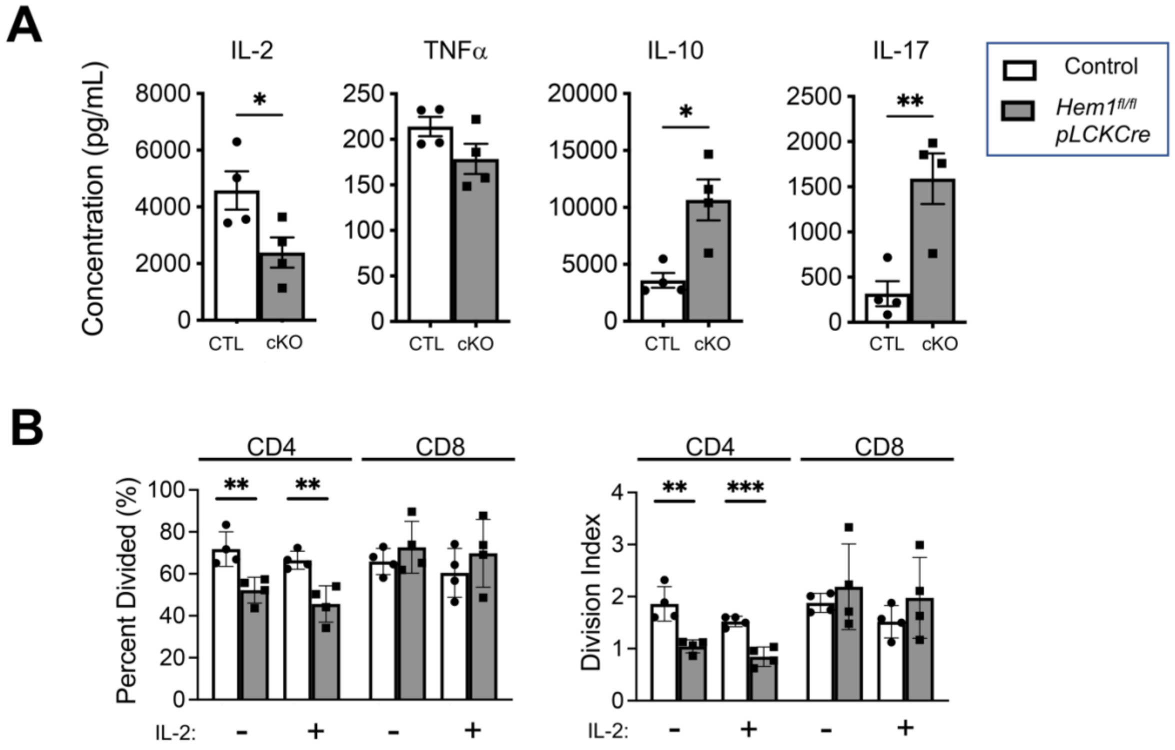

Supplemental Figure 6. T cell-specific deletion of *Hem1* results in altered cytokine production, and decreased cell proliferation is independent of decreased IL-2 levels.
(A) Purified T cells from 10-12-week-old *Hem1$^{fl/fl}$pLCKCre* mice and *Hem1$^{fl/fl}$* littermate controls were stimulated with anti-CD3 and anti-CD28 antibodies for 72 hours. Concentrations of cytokines in supernatant were measured by multiplex immunoassay. **(B)** Purified T cells from 39-43-week-old *Hem1$^{fl/fl}$pLCKCre* mice and *Hem1$^{fl/fl}$* littermate controls were stimulated with anti-CD3 and anti-CD28 antibodies with or without IL-2 supplementation (100ng/ml) for 72h. Proliferation was assessed with CFSE, and bar graphs represent the percent of cells that had undergone division. Each data point represents an individual mouse, n-4/group. Bar graphs represent mean ± SD and were analyzed via unpaired Student's t test. **P* < 0.05, ***P* < 0.01, ****P* < 0.001. Rel = relative.

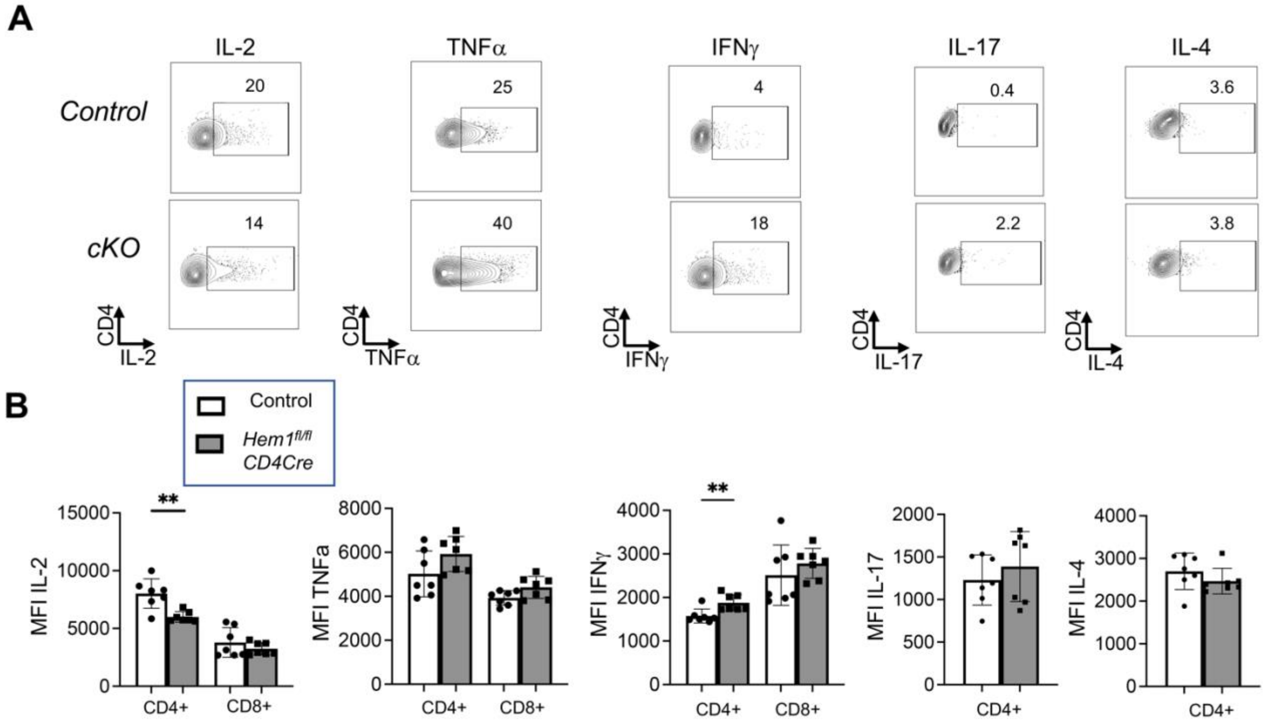

Supplemental Figure 7. T cell-specific deletion of *Hem1* results in altered cytokine production.

Purified T cells from splenocytes harvested from *Hem1$^{fl/fl}$CD4Cre* mice and *Hem1$^{fl/fl}$* littermate controls were stimulated with anti-CD3 and anti-CD28 antibodies for 72h followed by PMA and ionomycin stimulation for 5h. **(A)** Representative flow cytometric histograms of intracellular staining of the indicated cytokines. Cells first gated on lymphocytes, single cells, live cells, and $CD4^+$ cells. **(B)** Bar graphs represent MFI of indicated cytokines in $CD4^+$ and $CD8^+$ T cells. 11-15-week-old mice, n=7/group. Data representative of 2 or more independent experiments, each dot represents an individual mouse. Bar graphs represent mean ± SD and were analyzed via unpaired Student's t test. *$P < 0.05$, **$P < 0.01$, ***$P < 0.001$.

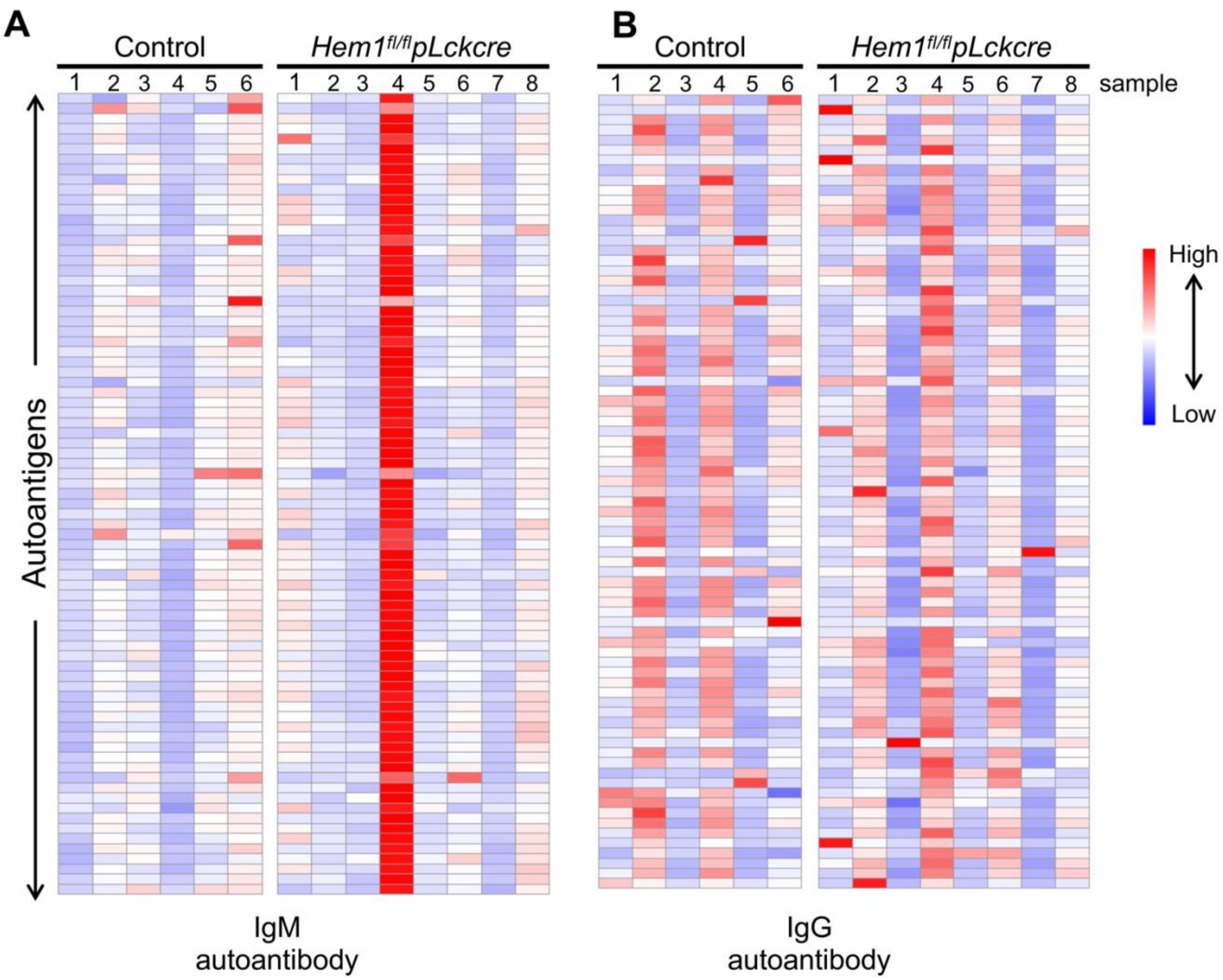

Supplemental Figure 8. T cell specific disruption of Hem1 does not result in increased autoantibody formation

Sera were collected from female *Hem1$^{fl/fl}$pLckCre* and control mice ages 49–56 weeks (n=6-8/group). Sera were then hybridized to an autoantigen microarray containing 128 antigens. Heatmaps depict antigen reactivity (antibody score) for **(A)** IgM and **(B)** IgG.

Supplemental Table 1. Comparison of T cell populations in human and mouse models of *Hem1* deficiency

	Human PID	Hem1$^{-/-}$	Hem1$^{fl/fl}$pLckCre	Hem1$^{fl/fl}$CD4Cre
CD4	↓ Low (Castro)	↓ Low	LN: ↓ Low (%)	LN, Spl: ↓ Low
CD8	↑ High (Castro)	↑ High (%), ↓ Low (#)	Spleen: ↓ Low (%)	LN, Spl: ↓ Low
$\alpha\beta$ T cells	n/a	↓ Low	↓ Low (%)	↓ Low
$\gamma\delta$ T cells	↑ High (Salzer)	↓ Low	↑ High (%)	↑ High (%)
Treg	↓ Low (Castro)<br>Normal (Cook)	↑ High (%)	↑ High (%)	↑ High (%)
Naive T cells				
CD4	↓ Low (Cook, Castro, Salzer)	↓ Low	↓ Low	↓ Low
CD8	↓ Low (Cook, Castro, Salzer)	↓ Low	↓ Low	↓ Low
EM T cells				
CD4	↑ High (Castro, Salzer)	↑ High	↑ High	↑ High
CD8	↑ Normal to high (Castro, Salzer)	↑ High	No difference	↑ High
Hepato-splenomegaly	Yes (Cook, Castro, Salzer)	Yes	No	No
Liver mineralization	Yes (Cook)	Yes	n/a	n/a

EM = effector memory

Supplemental Table 2. Comparison of T cell activation and effector function in human and mouse models of T cell specific *Hem1* deficiency

	Human PID	Mouse cKO model
CD69 expression	↓ Low (Castro, Cook, Salzer)	↓ Low (CD4, CD8)
CD25 expression	↓ Low (Castro, Cook, Salzer)	↓ Low (CD4, CD8)
Proliferation	↓ Low (Cook, Salzer)<br>No difference (Castro)	↓ Low (CD4, CD8)
Exhaustion marker expression	↑ High (Castro, Salzer)	↑ High
IS formation	↓ Low (Cook, Castro)	↓ Low
Cytokines		
IL-2	↓ Low (Cook)	↓ Low
IL-4	n/a	No difference
IL-10	↑ High (Castro)	↑ High
IL-17	n/a	↑ High
IFNγ	↑ High (Castro)	↑ High
TNFα	↓ Low (Cook)	↑ High
mTORC2 signaling	↓ Low (Cook, Salzer)	↑ High

cKO = conditional knockout

1. Avalos A, Tietsort JT, Suwankitwat N, Woods JD, Jackson SW, Christodoulou A, et al. Hem-1 regulates protective humoral immunity and limits autoantibody production in a B cell-specific manner. *JCI Insight.* 2022;7(9).
