# Supplementary figures and images for: Hem1 controls T cell activation, memory, and the regulated release of immunosuppressive and proinflammatory cytokines
Source: JCI Insight. 2025 Jul 8;10(16):e174235. doi: 10.1172/jci.insight.174235 (PMC12406723; doi:10.1172/jci.insight.174235)

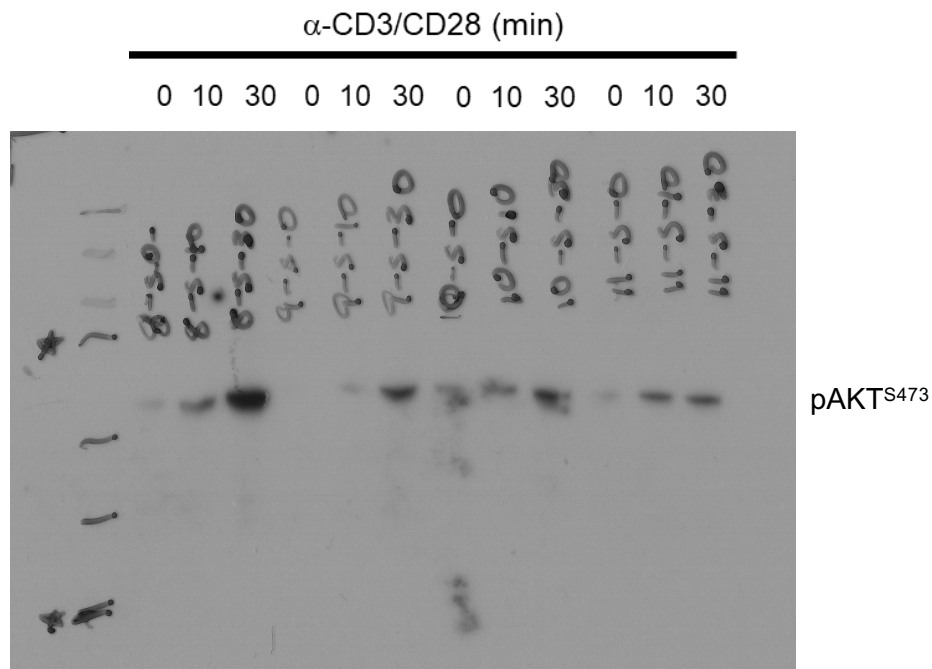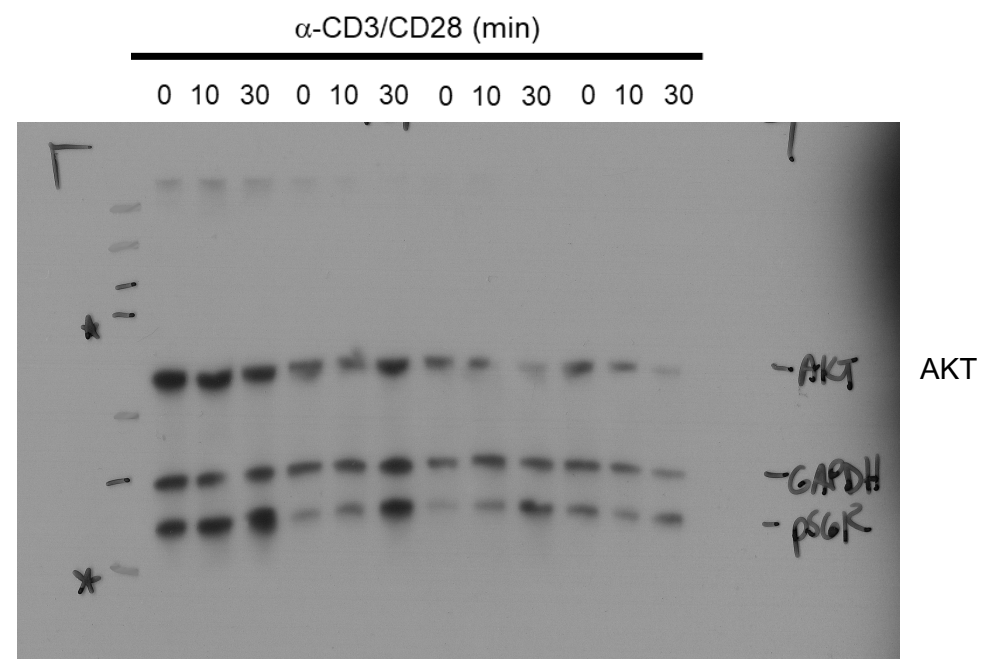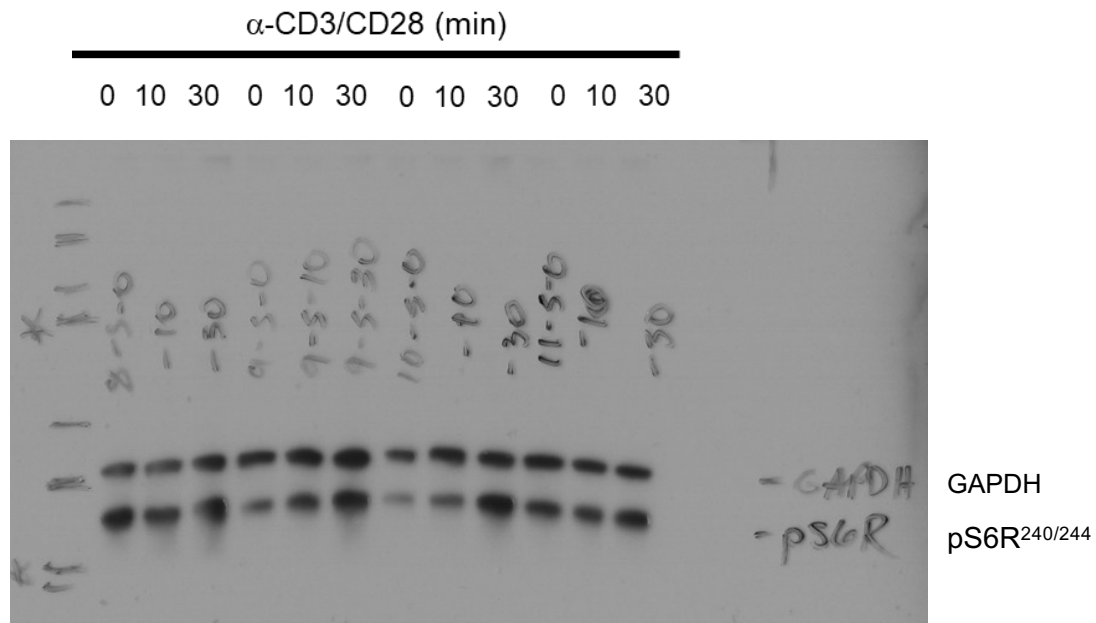

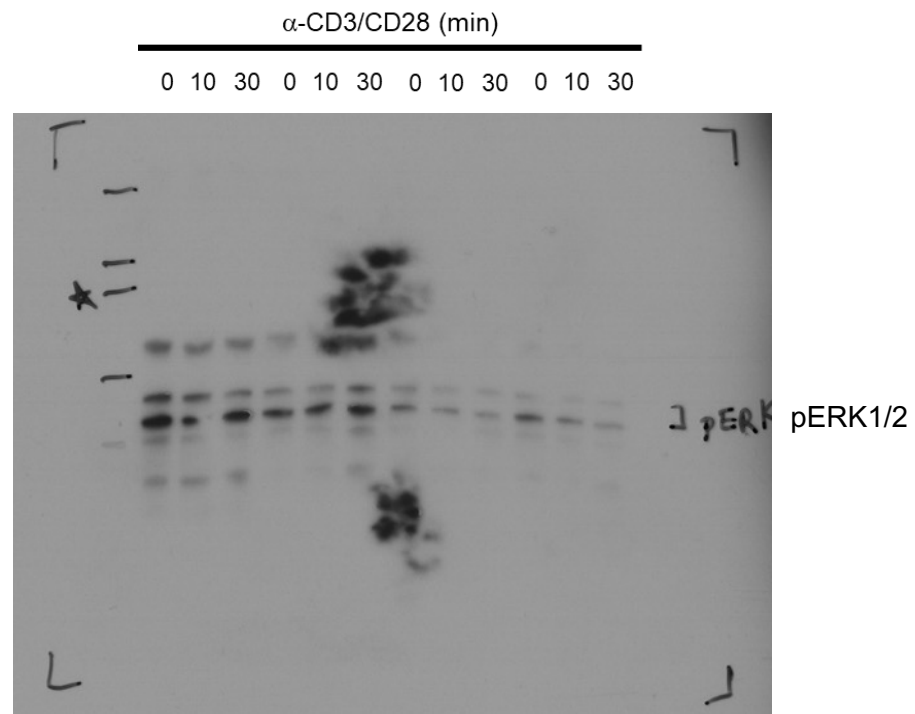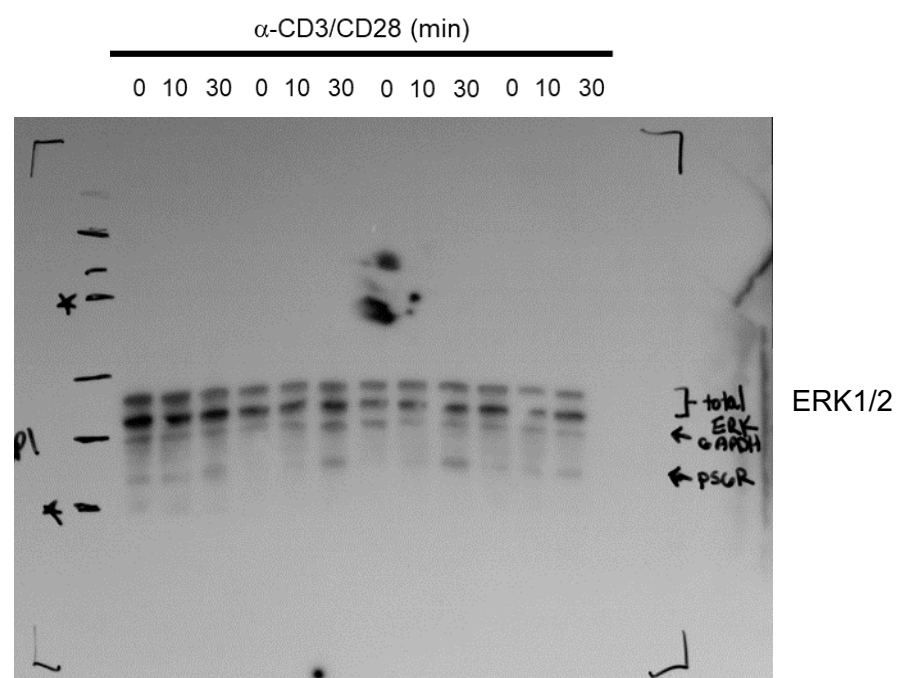

# Figure 8 blot

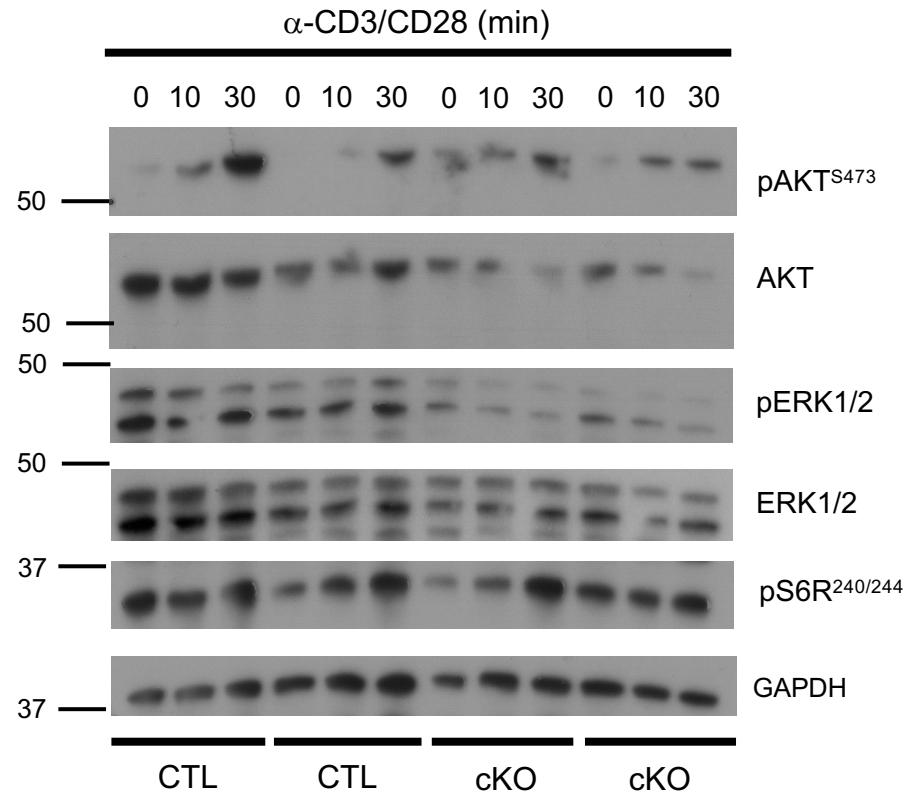

Supplement: Unedited blot and gel images [file jciinsight-10-174235-s124.pdf]
